# Phenotypic heterogeneity in lag reflects an evolutionarily stable bet-hedging strategy

**DOI:** 10.1101/2025.11.02.686100

**Published:** 2025-11-11

**Authors:** Abir George, Ned S. Wingreen, Gautam Reddy

**Affiliations:** Lewis-Sigler Institute for Integrative Genomics, Princeton University, Princeton, New Jersey 08544, USA; Lewis-Sigler Institute for Integrative Genomics, Princeton University, Princeton, New Jersey 08544, USA and Department of Molecular Biology, Princeton University, Princeton, New Jersey 08544, USA; Joseph Henry Laboratories of Physics, Princeton University, Princeton, New Jersey 08544, USA

## Abstract

Single-cell experiments in yeast reveal two distinct heritable phenotypes—‘arresters’ and ‘recoverers’—when a clonal population experiences a negative shift in its growth environment. Recoverers exhibit a variable yet finite lag before resuming growth in the new environment, whereas arresters remain in a non-growing, arrested state until more favorable conditions return. Although the diversification of individual cells into arresters and recoverers is a robust phenomenon, it remains unclear whether this coexistence constitutes an evolutionarily stable strategy. Here, we demonstrate that a heterogeneous strategy composed of both arrester and recoverer phenotypes maximizes long-term population fitness across a broad spectrum of growth-lag trade-offs. Our analysis employs a dynamic programming framework to identify the fitness-maximizing distribution of phenotypes for populations that stochastically switch between preferred and non-preferred environments. We propose a minimal model incorporating metabolism, growth, and enzyme allocation to explain the physiological origin of a power-law growth-lag trade-off that favors phenotypic heterogeneity. The theory predicts a nontrivial relationship between the fraction of recoverers and their lag time, which aligns with existing data from wild yeast strains, evolved isolates, and variations in pre-shift growth conditions. This relationship suggests an evolutionary ‘rheostat’-like mechanism that enables populations to rapidly adapt to changing environmental conditions.

## I. INTRODUCTION

Microbes typically live in environments characterized by rapid and unpredictable changes in nutrient availability, temperature, pH, and other critical factors. To persist and thrive under these uncertain conditions, microbes have evolved diverse adaptive strategies. These include mechanisms for physiological adaptation and gene regulation, as well as phenotypic heterogeneity – whereby multiple distinct phenotypes coexist within genetically identical populations. Notably, phenotypic heterogeneity can act as a bet-hedging mechanism, allowing microbial populations to hedge against environmental uncertainty, thus enhancing their long-term success. A well-known example of bet-hedging is the formation of antibiotictolerant cells in *Escherichia coli* [1–3]. Within a clonal population, a small fraction of cells grows very slowly, or not at all, which makes them more likely to survive antibiotics and other stressors. These ‘persister’ cells serve as a reservoir for repopulation once favorable conditions are restored. Bet-hedging through phenotypic diversification has been extensively explored in ecological and evolutionary theory, with numerous theoretical studies analyzing its implications [4–12].

Recent experiments in yeast have revealed a distinctive form of phenotypic heterogeneity in response to nutrient shifts. Upon a shift between environments with different nutrients, microbes undergo a period of slow or halted growth, termed the lag phase, during which cells express the machinery that enables growth in the new environment [13]. Yeast (including *Saccharomyces cerevisiae* and its relatives) display a lag period when transferred from a preferred carbon source like glucose to alternative nutrients such as maltose, galactose, or ethanol. Quantitative characterization of this lag phase has revealed considerable variability in the duration and dynamics of lag across natural yeast strains [14–20]: while some strains rapidly resume growth upon a shift, others display many hours of lag before gene expression can reorganize to resume growth.

**FIG. 1.**
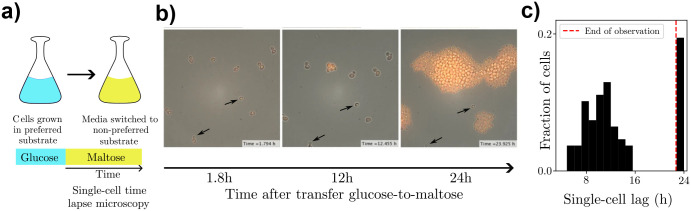
Heterogeneity in single-cell lag durations following a glucose-to-maltose shift. (a) Schematic of experimental protocol: yeast cells are pre-grown in glucose, then the media is switched to maltose where cells are tracked using time-lapse microscopy. (b) Time-lapse images of a yeast population following a sudden switch from glucose to maltose. MAL12-yECitrine fluorescence indicates growth resumption. Images taken at 1.8h, 12h, and 24h after the switch. Cells exhibit heterogeneous behavior in lag phase recovery, including a subpopulation that remains arrested (indicated by black arrows). Images reproduced from [17]. (c) Distribution of single-cell lag durations across the population. The red dashed line marks the end of the observation period (23.5 h), beyond which lag durations were not measured.

Beyond this population-level variability observed across strains, single-cell experiments have also found significant heterogeneity in lag. Individual cells from a clonal yeast population display marked variation in lag duration, ranging from immediate resumption of growth to prolonged growth arrest [14, 17]. A standard experimental protocol is to transfer isogenic yeast cells grown, for example, in glucose to maltose and track single-cell growth using time-lapse microscopy (Fig. 1). Cells show two distinct modes of phenotypic response: while some cells (termed ‘recoverers’) resume growth after a few hours, a fraction of cells (termed ‘arresters’) remain in a non-growing, arrested state for the entire duration (~24 hours) of the experiment (Fig. 1b,c). The arrested state is characterized by deformations of sub-cellular structures [14] and is reversible, that is, arresters resume growth when re-introduced into a glucose-rich environment. While this bimodal response is consistently observed across wild strains and evolved isolates as well as growth media and the time spent in the pre-shift environment, the ratios of recoverers to arresters and the distributions of lag durations within the recoverer subpopulation both vary substantially across these scenarios [17, 20]. A natural question is whether this coexistence of arrester and recoverer states reflects a stable evolutionary strategy.

If rapid recovery from lag entailed a negligible physiological cost (for example, in pre-shift growth rate), then we should expect cells to prefer a homogeneous strategy that recovers as quickly as possible. However, the observation of an arrested state suggests that cells face intrinsic limitations when adapting to maltose or other less-preferred carbon sources. Extensive experimental work has characterized trade-offs between growth and adaptability in microbes [14, 16, 18, 21–26]. When such trade-offs are present, the coexistence of distinct phenotypes can emerge as an evolutionarily stable strategy [14, 23–25, 27]. Thus, one possible explanation for the observed coexistence is that individual yeast cells are constrained by some nontrivial physiological trade-off that prevents them from simultaneously achieving rapid pre-shift growth and rapid post-shift recovery. This hypothesis is supported by measurements which show that arresters grow faster than recoverers in the pre-shift environment [14].

To determine the evolutionarily stable strategy in the presence of such a growth-lag trade-off, we leverage a novel optimization framework based on dynamic programming. The framework leads to a general decision-making principle that can be used to find fitness-maximizing bet-hedging strategies for a population fluctuating between a preferred and a non-preferred environment. We show that, depending on environmental statistics and the physiological trade-off, the population either adopts a bet-hedging strategy composed of two “specialist” phenotypes (arresters and recoverers) that respectively are well-suited for growth and rapid recovery, or picks a “generalist” phenotype that balances both traits. We propose a minimal metabolic model that explains the physiological origin of a power-law-like growth-lag trade-off. Furthermore, the model predicts that populations experiencing different environmental statistics follow a characteristic relationship between the ratio of recoverers and arresters, and the lag duration of recoverers. We test this prediction using existing experimental data, which hints at an underlying evolutionary mechanism that enables rapid adaptation to changes in environmental statistics.

## II. THEORETICAL FRAMEWORK

To quantify the long-term fitness benefits of phenotypic heterogeneity, we developed a cellular decision-making framework based on dynamic programming [28]. We first describe the general framework before specializing to the growth-lag scenario. Another application of this framework to a growth-death scenario is presented in Appendix B4.

Consider a microbial population where cells adopt one of K phenotypic states, labeled by k, each of which is characterized by its growth rate γ(s,k) in state s. The state s can represent, for example, the time since a nutrient switch, the concentration of a nutrient, or any history-dependent quantity tracked by the cell. The instantaneous growth rate of a population of N cells at time t is

(1) $$\frac{1}{N}\frac{dN}{dt}=\sum_{k=1}^{K}\phi_{k}\left(t\right)\gamma \left(s\left(t\right),k\right)\equiv \overset{‾}{\gamma }\left(s\left(t\right),\phi \left(t\right)\right),$$

where $\phi_{k}$ is the fraction of cells with phenotype k and $\phi =\left(\phi_{1},\phi_{2},\ldots ,\phi_{K}\right)$ is the phenotype distribution. The long-term growth rate Γ of the population is then

(2) $$\Gamma =\underset{T\to \infty }{\text{lim}}\frac{1}{T}\int_{0}^{T}\left\langle \overset{‾}{\gamma }\left(s\left(t\right),\phi \left(t\right)\right)\right\rangle dt,$$

where the angled brackets denote an expectation over changes in state.

**FIG. 2.**
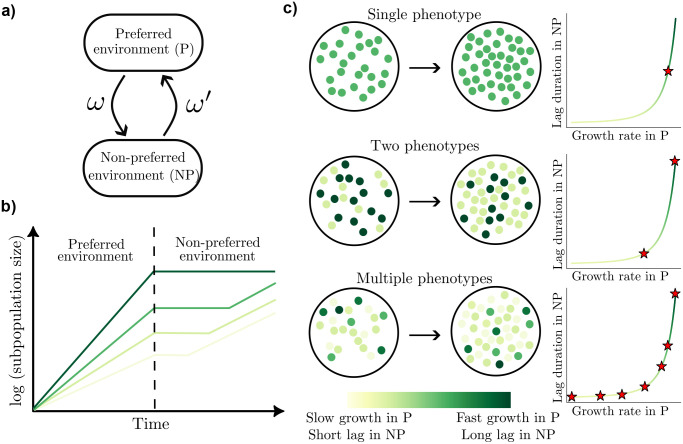
Phenotypic strategies in fluctuating environments. (a) Model of environmental switching with transitions between a preferred (P) and a non-preferred (NP) environment occurring at rates ω and $\omega^{\prime }$, respectively. (b) Growth dynamics of pure phenotype subpopulations in a fluctuating environment. Distinct phenotypes grow at different rates in the preferred environment (P) and experience varying lag durations upon transition to the non-preferred environment (NP), with a trade-off between growth rate in P and lag duration in NP. Cells that grow slowly in P recover more quickly and resume growth earlier in NP. As a result, these phenotypes increase in relative abundance during NP. (c) Schematic showing possible strategies for a population of cells: a single phenotype (top), two phenotypes (middle), or multiple phenotypes (bottom). Disks represent individual cells colored by their phenotype, corresponding to a position on the growth-lag trade-off curve (right). Red stars indicate the phenotypes that comprise the strategy.

We focus on populations that transition repeatedly between preferred (P) and non-preferred (NP) environments, such as yeast shifting between glucose and maltose. Importantly, we assume that in P the population can rapidly reorganize gene expression and thereby adjust its phenotype distribution, whereas cells remain phenotypically locked upon the switch to NP. This environmental switching model is schematically illustrated in Fig. 2a,b. Fluctuating environments will select for phenotype distributions that best balance overall population growth in P and NP. A key conceptual question is when and whether this best population-level strategy consists of one, two, or multiple phenotypes (Fig. 2c).

Our objective is thus to identify the optimal phenotype distribution $\phi^{*}$ in P that maximizes the long-term growth rate Γ in the face of environmental fluctuations. Given state s, and our assumption of rapid phenotype adjustment in P, we show that

(3) $$\phi^{*}(s)=\text{arg}\;\underset{\phi }{\text{max}}\left\{{\overset{‾}{\gamma }}_{\text{P}}(s,\phi )+\omega (s)\left\langle \int_{0}^{\infty }d\tau \;\Pi (\tau )\left(\int_{0}^{\tau }dt\;{\overset{‾}{\gamma }}_{\text{NP}}(s(t),\phi (t))\right)\right\rangle \right\},$$

where Π(τ) is the probability density of the duration τ spent in NP before switching back to P,ω(s) is the instantaneous rate of switching from P to NP and we have included the subscripts P and NP to $\overset{‾}{\gamma }$ for clarity. Equation 3 has an intuitive interpretation: the optimal strategy balances the instantaneous growth rate in P (first term) and the expected long-term growth rate if the environment were to transition to NP, scaled by the probability that the transition occurs (second term).

## III. RESULTS

### Optimal strategies given a growth-lag trade-off.

In our growth-lag scenario, cells with phenotype k have a growth rate $\gamma_{k}$ in P and a lag duration $\ell_{k}$ in NP with no growth during lag. After recovering from lag, cells grow in NP at a common rate $\gamma^{\prime }$. Phenotypes experience a growth-lag trade-off: phenotypes that grow faster (slower) in P exhibit longer (shorter) lag durations in NP (Fig. 2b). To incorporate the effect of lag, the state s(t) in NP tracks the time since the most recent transition from P to NP. For simplicity, we drop any dependence of ϕ on s in P, noting however that the results also apply to scenarios where $\phi^{*}$ depends on the nutrient in P or historical information that could be used to anticipate a switch. The optimization objective then becomes

(4) $$\phi^{*}=\text{arg}\;\underset{\phi }{\text{max}}\left\{{\overset{‾}{\gamma }}_{\text{P}}\left(\phi \right)+\omega \int_{0}^{\infty }d\tau \;\Pi \left(\tau \right)\int_{0}^{\tau }dt\;{\overset{‾}{\gamma }}_{\text{NP}}\left(t,\phi \left(t\right)\right)\right\},$$

from which we derive the specific optimization objective

(5) $$\phi^{*}=\text{arg}\;\underset{\phi }{\text{max}}\left\{\sum_{k=1}^{K}\phi_{k}\gamma_{k}+\omega \int_{0}^{\infty }dte^{-\omega^{\prime }t}\frac{\sum_{k=1}^{K}\phi_{k}\gamma^{\prime }\Theta \left(t-\ell_{k}\right)e^{\gamma^{\prime }{\left[t-\ell_{k}\right]}_{+}}}{\sum_{k=1}^{K}\phi_{k}e^{\gamma^{\prime }{\left[t-\ell_{k}\right]}_{+}}}\right\},$$

where $\omega^{\prime }$ is an assumed constant rate of switching from NP back to P so that $\Pi (\tau )=\omega^{\prime }e^{-\omega^{\prime }\tau },\Theta \left(t-\ell_{i}\right)$ is the Heaviside function and ${\left[t-\ell_{i}\right]}_{+}=\text{max}\left(0,t-\ell_{i}\right)$. Integrating Eq. 5 by parts, the objective function can be simplified to

(6) $$\phi^{*}=\text{arg}\;\underset{\phi }{\text{max}}\left\{\sum_{k=1}^{K}\phi_{k}{\tilde{\gamma }}_{k}+\int_{0}^{\infty }dt^{\prime }e^{-\frac{t^{\prime }}{\eta }}\text{log}\;\left[\sum_{k=1}^{K}\phi_{k}e^{\left[t^{\prime }-\ell_{k}^{\prime }\right]+}\right]\right\},$$

where ${\tilde{\gamma }}_{k}=\gamma_{k}/\omega$ and $\ell_{k}^{\prime }=\gamma^{\prime }\ell_{k}$ represent normalized growth rates and lag durations respectively, and $\eta =\gamma^{\prime }/\omega^{\prime }$ is the average number of generations of a no-lag subpopulation in NP.

A key factor determining a population’s optimal strategy in a fluctuating environment is the form of the growth-lag trade-off. To capture the range of possible behaviors, we consider a family of growth-lag trade-offs:

(7) $$\gamma_{\text{P}}\left(\ell \right)=\gamma_{\text{max}}-\left(\gamma_{\text{max}}-\gamma_{\text{min}}\right){\left(\frac{\ell_{\text{min}}}{\ell }\right)}^{\alpha },$$

where lag $\ell \geq \ell_{\text{min}}$, and the growth rate in P lies in the range $\gamma_{\text{min}}\leq \gamma_{\text{P}}\leq \gamma_{\text{max}}$. The parameter α defines a family of trade-off curves, with larger α corresponding to larger $\gamma_{\text{P}}$ for the same lag ℓ. This parametric form always includes phenotypes that have maximum growth in P at the cost of infinite lag (ℓ=∞) in NP.

### Numerical result: at most two phenotypes, with one arrester.

**FIG. 3.**
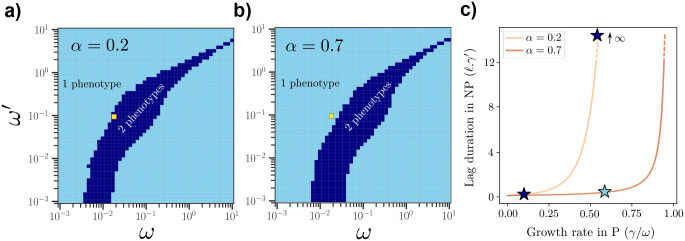
Optimal phenotypic strategies consist of at most two distinct phenotypes. (a, b) Phase diagrams of the optimal number of phenotypes over the space of environmental switching rates, ω and $\omega^{\prime }$, for fixed trade-off curves parameterized by α=0.2 (a) and α=0.7 (b). Light blue regions correspond to a single optimal “generalist” phenotype, while dark blue regions correspond to two coexisting “specialists”, an arrester and a recoverer. (c) Different trade-off curves between growth rate in P and lag duration in NP from A and B, with optimal phenotypes for the $\left(\omega ,\omega^{\prime }\right)$ values indicated by yellow squares. For increasing α, the optimal strategy transitions from a two-phenotype (specialist) solution (dark blue stars) to a single-phenotype (generalist) solution (light blue stars).

We numerically computed the optimal phenotype distribution over a continuous set of phenotypes constrained by the above growth-lag trade-off curve (Eq. 7). Although one might have expected the optimal strategy to exploit a broad range of phenotypes, we found that the optimal distribution contains at most two distinct phenotypes. This restriction to no more than two phenotypes applies across the whole parameter space defined by the environmental transition rates $\left(\omega ,\omega^{\prime }\right)$, as illustrated in Fig. 3a,b.

Within the two-phenotype regime, the optimal strategy consistently comprises one phenotype with finite lag – a recoverer – and another phenotype with infinite lag (ℓ=∞) – an arrester. The recoverer is adapted to resume growth in the non-preferred environment (NP), while the arrester is specialized for maximal growth in the preferred environment (P). The optimal strategy assigns a nonzero probability to the arrester phenotype, even though it never grows in NP. This is perhaps a surprising result: it implies that under certain environmental conditions, it is beneficial for a population to maintain a subpopulation that entirely forfeits the ability to grow in one environment in order to maximize performance in the other, even though both environments can support growth. Similarly, the recoverer phenotype sacrifices growth in P in order to perform better in NP. The two phenotypes in this bet-hedging strategy can thus be viewed as *specialists*, each appropriately suited for a distinct environmental state.

It is clear that the phase diagram is re-entrant, that is, for increasing $\omega /\omega^{\prime }$, the optimal strategy goes from generalist to specialist, and back to generalist. For small $\omega /\omega^{\prime }$, the generalist strategy corresponds to having only the arrester in the population since the environment is mostly P. By contrast, for $\omega /\omega^{\prime }\approx 1$, the population should optimally adopt a specialist strategy (with deviations with respect to this ratio for $\omega ,\omega^{\prime }\ll 1$ and $\omega ,\omega^{\prime }\gg 1$). When $\omega /\omega^{\prime }$ increases beyond this, the optimal strategy reverts to a generalist strategy with a recoverer phenotype. This makes sense as the population spends more time in NP and the recoverer phenotype is able to maximally contribute to the population growth.

As shown in Fig. 3c, varying the shape of the growth-lag trade-off via the power-law exponent α can qualitatively change the optimal strategy for fixed $\left(\omega ,\omega^{\prime }\right)$. For example, for the $\left(\omega ,\omega^{\prime }\right)$ values in the yellow squares in Fig. 3a,b, at low α the optimal strategy is two specialists (one being an arrester), while at higher α the optimal strategy is a single *generalist* phenotype that balances growth in P and lag in NP.

### Phenotypic distributions with a few discrete phenotypes are optimal.

The observation that the optimal phenotype distribution contains a few (here, at most two) discrete phenotypes can be explained in the special case when the time spent in the non-preferred environment (NP) is fixed at a constant value t. In this case, the optimization objective in Eq. 6 reduces to (with $t^{\prime }=\gamma^{\prime }t$)

(8) $$\phi^{*}=\text{arg}\;\underset{\phi }{\text{max}}\left\{\sum_{k=1}^{K}\phi_{k}{\tilde{\gamma }}_{k}+\text{log}\;\left(\sum_{k=1}^{K}\phi_{k}e^{{\left[t^{\prime }-\ell_{k}^{\prime }\right]}_{+}}\right)\right\},$$

with the constraints $\sum_{k=1}^{K}\phi_{k}=1$ and $\phi_{k}\geq 0$ for all k. The Karush-Kuhn-Tucker (KKT) theorem provides a set of conditions satisfied by $\phi^{*}$ subject to these constraints. The KKT conditions imply that the growth rates and lag durations of any pair of phenotypes (indexed by m and n) with nonzero probability are related according to

(9) $$\frac{e^{{\left[t^{\prime }-\ell_{m}^{\prime }\right]}_{+}}-e^{{\left[t^{\prime }-\ell_{n}^{\prime }\right]}_{+}}}{{\tilde{\gamma }}_{m}-{\tilde{\gamma }}_{n}}=C,$$

for some constant C. This restrictive condition may be true for a continuous set of phenotypes in certain special scenarios, for example, when $\gamma_{P}(\ell )\propto e^{-\gamma^{\prime }\ell }$, but that will not be true in most scenarios, including for the family of growth-lag trade-offs in Eq. 7. Some geometric intuition can be obtained by noting that solutions $\phi^{*}$ for an objective of the form Eq. 8 will lie on the vertices of a region formed by the intersection of a hyperplane with normal vector $\gamma =\left(\gamma_{1},\gamma_{2},\ldots ,\gamma_{K}\right)$ and the probability simplex. These vertices are points with nonzero probability for at most two phenotypes. For complex dwell time distributions in NP, it is possible that the optimal phenotypic distribution has more than two phenotypes. However, we consistently find at most two phenotypes for the exponentially distributed dwell times considered in our numerical simulations, presumably because this distribution has a single typical timescale.

### The geometry of the growth-lag trade-off determines whether a generalist or specialist strategy is preferred.

When is a single generalist phenotype favored over a mixture of two specialist phenotypes? Optimizing Eq. 4 for the single best phenotype solution, the lag $\ell_{\text{g}}$ of the generalist phenotype (provided it is finite) satisfies $d\gamma_{\text{P}}/{\left.d\ell \right|}_{\ell_{\text{g}}}=\omega \gamma^{\prime }F\left(\ell_{\text{g}}\right)$, where $F(\ell )=\int_{\ell }^{\infty }d\tau \Pi (\tau )$ for general growth-lag trade-offs $\gamma_{\text{P}}$ and dwell time distributions Π(τ) (Appendix D). Considering phenotypes in the neighborhood of $\ell_{\text{g}}$, we find that a mixed strategy is preferred if (Appendix D)

(10) $$-{\left.\frac{d^{2}\gamma_{\text{P}}}{d\ell^{2}}\right|}_{\ell_{\text{g}}}<\omega \gamma^{\prime }\left(\Pi \left(\ell_{\text{g}}\right)+\gamma^{\prime }F\left(\ell_{\text{g}}\right)\right).$$

This condition implies that the curvature of the growth-lag trade-off around the generalist solution determines whether a pure or a mixed strategy is favorable. Moreover, the solutions on the two sides of the transition point (i.e., when equality holds in Eq. 10) are discontinuous: extreme specialists are preferred whenever a mixed strategy is optimal (Appendix D).

### The optimality of the arrester phenotype.

A consistent observation in our numerical simulations is the selection of a perfect arrester phenotype (with infinite lag) whenever a mixed strategy is preferred. The selection of this arrester phenotype can be explained by a heuristic argument based on how the growth-lag trade-off scales at long lags and the tail probabilities of dwell times in NP (see Appendix E1 for a longer discussion). Since the distribution of dwell times in NP decays exponentially, the marginal cost of increasing the lag of the arrester phenotype also falls off at the same exponential rate. On the other hand, the marginal benefit in pre-shift growth rate declines more slowly according to a power-law given by Eq. 7. Thus, for long lags, any increase in lag of the arrester delivers a net positive marginal benefit, implying that it is always beneficial to increase the lag indefinitely.

### A minimal metabolic model reproduces a power-law-like trade-off between pre-shift growth rate and post-shift lag duration.

**FIG. 4.**
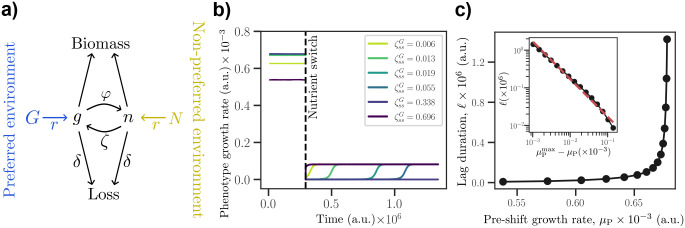
A minimal metabolic model with a growth-lag trade-off. (a) Schematic showing two central metabolites g and n where enzymes φ catalyze g→n, and enzymes ζ catalyze n→g. In the preferred environment, nutrient G is present and N is absent. Allocating enzymes to high φ achieves a high growth rate in this environment. However, after a switch to the non-preferred environment, pre-existing high φ prolongs the reaction g→n, which delays net flux reversal, producing a lag in growth until φ falls and ζ rises. (b) Time courses around the nutrient switch (vertical dashed line) showing the growth rates of different enzyme-allocation phenotypes. We define “lag” to end when the post-shift growth rate exceeds half the steady-state growth rate $\mu_{\text{NP}}$ in the non-preferred environment. (c) Growth-lag trade-off curve generated by varying pre-shift enzyme allocation, keeping $\varphi_{\text{ss}}^{G}=1$ fixed while decreasing $\zeta_{\text{ss}}^{G}$ from 1 to 10^−2.2^. This monotonically increases pre-shift growth rate $\mu_{\text{P}}$ but prolongs post-shift lag ℓ. The inset shows the predicted power-law scaling with exponent −1 (red dashed line) for ν=2. Details of the model are given in Appendix G and the parameter values are listed in Table I.

How might a power-law-like growth-lag trade-off arise? Studies in bacteria [21, 22] reveal that cells primed to grow quickly on a glycolytic nutrient experience a lag upon switching to a gluconeogenic nutrient. Guided by these observations and previous analysis, we developed a minimal metabolic model (Fig. 4) that results in a power-law-like trade-off between pre-shift growth rates and post-shift lag durations. In a model where nutrients are metabolized by independent pathways, it is not clear why a strong growth-lag trade-off should exist. The allocation of catalytic enzymes for the two pathways could be independent, allowing growth and lag to be decoupled and separately optimized.

In our model, two nutrients are processed along the same pathway, but the metabolic flux is controlled by catalytic enzymes that favor opposing directions (Fig. 4a). A key feature of this model is that after a nutrient switch, the imbalance in enzyme abundances which enabled flux in one direction must be reversed to allow flux in the other. This reversal in turn requires biomass production, which is slow until flux can be reversed. The coupling between flux reversal and biomass production consequently leads to a long lag. Thus, this minimal model is sufficient to obtain a growth-lag trade-off.

We quantify growth in the preferred environment (P) by the steady-state growth rate $\mu_{\text{P}}$ and define lag, ℓ, in the non-preferred environment (NP) as the time for resumed growth to exceed half the NP steady-state growth rate $\mu_{\text{NP}}$ (i.e. ℓ is the first-passage time for post-shift growth rate $\geq \mu_{\text{NP}}/2$). The model tracks two central metabolites, g and n, interconverted by two opposing enzyme activities: φ catalyzes g→n and ζ catalyzes n→g as shown in Fig. 4a. Metabolic fluxes are Michaelis-Menten, growth rate via biomass production μ is a cooperative function of g and n, and enzyme levels relax toward environment-specific set points via

$$\frac{d\varphi }{dt}=\mu \left(\varphi_{\text{ss}}^{(\text{nutrient)}}-\varphi \right),\frac{d\zeta }{dt}=\mu \left(\zeta_{\text{ss}}^{(\text{nutrient)}}-\zeta \right),$$

where the instantaneous growth rate is $\mu ={\left(\frac{n}{n+K_{\text{BM}}}\right)}^{\nu }\times {\left(\frac{g}{g+K_{\text{BM}}}\right)}^{\nu }$, and $\varphi_{\text{ss}}^{\text{(nutrient)}}$ and $\zeta_{\text{ss}}^{\text{(nutrient)}}$ are the steady-state concentrations of enzymes φ and ζ for a specified nutrient, respectively. For simplicity, the system is taken to be symmetric with respect to the two metabolites, except for the externally supplied nutrients. The rate constant r governs the influx into metabolites g and n, contributing rG and rN from G and N, respectively. We initialize the system at steady state in P with G at a fixed non-zero concentration $G_{\text{P}}$ and N=0, then impose an instantaneous environmental switch to NP by changing nutrient availability so that G=0 and N has non-zero concentration $N_{\text{NP}}<G_{\text{P}}$.

After the nutrient switch, high pre-existing φ suppresses the now-desirable net flux from n→g and leads to excess accumulation of n. This depletes cellular resources and delays the reversal of net flux. Slow biomass production gradually rebalances the enzymes, decreasing φ and increasing ζ, allowing the growth rate μ to rise to its steady-state value in NP. This rise time is an effective post-shift lag.

For each value of $\varphi_{\text{ss}}^{G}$, we vary $\zeta_{\text{ss}}^{G}$ to obtain a growth-lag trade-off. The values of $\varphi_{\text{ss}}^{N}$ and $\zeta_{\text{ss}}^{N}$ are kept fixed. As described below, we find that the metabolic model operates in at least three qualitatively distinct regimes for different values of $\varphi_{\text{ss}}^{G}$ depending on how the system produces new enzymes ζ after the switch from P to NP. Importantly, we obtain a power-law growth-lag trade-off for intermediate values of $\varphi_{\text{ss}}^{G}$. In Fig. 4b, we show specific time courses of the growth rate for different enzyme allocations (recoverer-like versus arrester-like) in this intermediate regime of $\varphi_{\text{ss}}^{G}$.

First, when $\varphi_{\text{ss}}^{G}$ is sufficiently small (and pre-shift growth is minimal), the depletion of g is gradual such that there is some growth (and thus production of ζ) for a short period after the switch from P to NP. The brief production of ζ is sufficient to avoid a prolonged lag phase as any non-zero ζ helps reverse the flux from n→g, promoting growth and subsequent production of ζ. That is, the system avoids a lag phase at the expense of minimal pre-shift growth.

Second, when $\varphi_{\text{ss}}^{G}$ is sufficiently large, the value of $\zeta_{\text{ss}}^{G}$ that maximizes growth in P is non-zero (Fig. S4). This is because a large $\varphi_{\text{ss}}^{G}$ rapidly depletes g even with influx from G in P. It is beneficial to oppose some of the flux from g→n via ζ to maintain a pool of g and n and promote growth. The pre-shift growth rate $\mu_{\text{P}}$ decreases quadratically with small deviations of $\Delta \zeta_{\text{ss}}^{G}$ from the growth-optimal allocation, that is, $\Delta \mu_{\text{P}}\sim -{\left(\Delta \zeta_{\text{ss}}^{G}\right)}^{2}$. Since $\zeta_{\text{ss}}^{G}$ is non-zero, the post-shift lag ℓ is finite even when pre-shift growth $\mu_{\text{P}}$ is maximal and ℓ decreases linearly with increasing $\Delta \zeta_{\text{ss}}^{G}:\Delta \ell \sim -\Delta \zeta_{\text{ss}}^{G}$. These two relations then lead to a quadratic relationship $\Delta \mu_{\text{P}}\sim -\Delta \ell^{2}$ near the growth-optimal $\zeta_{\text{ss}}^{G}$ for a given $\varphi_{\text{ss}}^{G}$. While the lag at maximal growth is finite, maintaining a large pool of enzymes $\varphi_{\text{ss}}^{G}$ and $\zeta_{\text{ss}}^{G}$ would incur a significant energetic cost. That is, the system could optimize pre-shift growth and still have finite lag after the switch but only by paying the energetic and resource cost of simultaneous expression of φ and ζ, which would lead to futile catalytic cycles [22].

Third, for intermediate values of $\varphi_{\text{ss}}^{G}$, the growth-optimal allocation is $\zeta_{\text{ss}}^{G}=0$ (Fig. S4). The rapid depletion of g after the switch and the lack of any conversion from n→g leads to infinite lag when $\zeta_{\text{ss}}^{G}=0$. Any small, non-zero $\zeta_{\text{ss}}^{G}$ decreases growth rate linearly, $\Delta \mu_{\text{P}}\sim -\zeta_{\text{ss}}^{G}$. Importantly, we show analytically that $\ell \sim {\left(\zeta_{\text{ss}}^{G}\right)}^{1-\nu }$ to first order in $\zeta_{\text{ss}}^{G}$ (for ν>1). This relation arises from the slow production of ζ after the switch. The two relationships, $\Delta \mu_{\text{P}}\sim -\zeta_{\text{ss}}^{G}$ and $\ell \sim {\left(\zeta_{\text{ss}}^{G}\right)}^{1-\nu }$, together imply a power-law-like growth-lag trade-off $\Delta \mu_{\text{P}}\sim -\ell^{-\alpha }$ with exponent $\alpha =(\nu -1{)}^{-1}$ (see Appendix G for more details). This predicted power-law relationship was verified in simulations (Fig. 4c).

### Theory predicts a relationship between the fraction of recoverers and their lag.

Our theory implies that for every choice of environmental statistics (specified by ω and $\omega^{\prime }$ in our model), there is a unique phenotype distribution that maximizes long-term growth. In other words, optimality requires that different features of the phenotype distribution (such as the fraction of recoverers and their lag) are not independent but rather co-vary with variations in environmental statistics. To derive these constraints, we now consider a simplified model where the phenotype distribution is restricted to at most two phenotypes. In this model, the phenotype distribution is parameterized by two quantities: the lag of the recoverer (denoted ℓ) and the fraction of recoverers (denoted ϕ). The remaining fraction 1-ϕ of arresters has infinite lag. For such a two-phenotype system, cells can select ϕ in P by stochastically switching between recoverer and arrester phenotypes. The difference in pre-shift growth rates between the arrester and recoverer phenotypes and the switching rates fix ϕ at steady-state (Appendix F).

Optimality of the phenotype distribution predicts that ℓ and ϕ co-vary according to a nontrivial relationship across different environmental statistics. For exponential dwell times in NP and the growth-lag trade-off in Eq. 7, we derived an analytical relationship between the optimal fraction $\phi^{*}$ of recoverers and their optimal lag $\ell^{*}$ (for details see Appendix E2). Whenever $0<\phi^{*}<1$, the relationship is given by

(11) γ′ℓ*α=ηF122,1η+1;1η+2;-1-ϕ*ϕ*ϕ*(1+η)F121,1η;1η+1;-1-ϕ*ϕ*,

where F12 is a hypergeometric function and recall that $\eta =\gamma^{\prime }/\omega^{\prime }$. As $\phi^{*}\to 1$, we have $\gamma^{\prime }\ell^{*}/\alpha =\eta /(1+\eta )$ and as $\phi^{*}\to 0,\gamma^{\prime }\ell^{*}/\alpha =\text{min}\{1,\eta \}$, which delineate the two boundaries of the coexistence region.

**FIG. 5.**
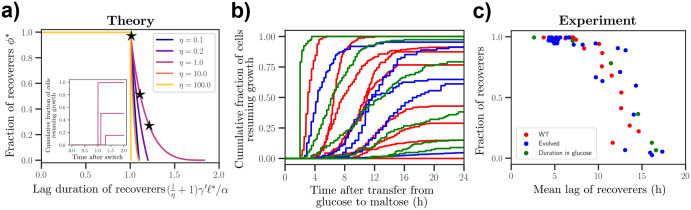
Comparison of theoretical predictions and experimental data for fraction of recoverers versus lag duration. (a) Theoretical relationship between the optimal fraction of recoverers and their lag duration for different values of $\eta =\gamma^{\prime }/\omega^{\prime }$. The lag duration is rescaled using parameters $\eta ,\gamma^{\prime }$ and α. Inset – Theoretical curves showing the single-cell lag duration after a switch from the preferred environment to the non-preferred environment. Different curves correspond to different points (black stars) on curve for η=1. Units for the x-axis match that of the main figure. (b) Experimental measurements of yeast single-cell lag duration after a switch from glucose to maltose, recorded via time-lapse microscopy. Each curve corresponds to the cumulative fraction of cells resuming growth after the switch. Red curves represent measurements for different wild strains, blue curves represent measurements for evolved isolates that experienced repeated cycling of glucose-maltose media, and green curves correspond to populations that experienced different pre-shift durations in glucose. Only a subset of these traces is shown here due to limited space. See Fig. S2 for more. Data from Ref. [15, 16]. (c) Experimental plot of the fraction of recoverers against the mean lag duration of the recoverers. Red, blue, and green dots represent, respectively, wild strains, evolved isolates, and variations based on pre-shift durations in glucose.

Figure 5a illustrates the predicted relationship between the optimal fraction of recoverers $\left(\phi^{*}\right)$ and their optimal lag $\left(\ell^{*}\right)$ for different values of η. We obtain a family of curves that show a consistent inverse relationship: $\ell^{*}$ initially increases while maintaining a single recoverer population $\left(\phi^{*}=1\right)$ with decreasing switching rate ω from P to NP. For a sufficiently small value of ω, the population splits into two phenotypes $\left(0<\phi^{*}<1\right)$ while the lag of the recoverer continues to grow until the population again consists of a single phenotype $\left(\phi^{*}=0\right)$. The ratio between the values of $\ell^{*}$ at the two boundaries of the coexistence region, $\phi^{*}\to 0$ and $\phi^{*}\to 1$, is at most two (achieved when η=1), imposing a constraint on the possible shapes in this family of curves.

An important point is that the relationship between the rescaled lag (in units of $\alpha /\gamma^{\prime }$) and fraction of recoverers is only mediated by the parameter η (the expected number of generations in NP of a no-lag phenotype). It is agnostic to factors such as genetic variability among different strains and dwell time distributions in the preferred environment, making it broadly applicable across diverse experimental conditions and yeast strains. We can thus quantitatively test this prediction for various environmental conditions and genetically distinct yeast populations using existing datasets.

### Experimental evidence for a relationship between fraction of recoverers and their lag.

We analyzed experimental data from single-cell measurements of yeast populations undergoing a glucose-to-maltose transition (Fig. 5b). These data report the cumulative fraction of cells resuming growth after a switch from media containing glucose to media containing maltose for up to 24 hours after the switch [16]. In Fig. 5b, each individual red curve represents lag measurements for a different wild strain of yeast, blue curves represent measurements taken for evolved isolates of a long-lag strain in parallel cultures after experiencing 6–8 cycles of alternating glucose and maltose-containing media, and green curves represent measurements for populations that experienced different glucose pre-growth durations. From the data, we obtained distributions of lag durations for each tracked population.

A consistent feature is the bimodality of these distributions, delineating two distinct subpopulations of recoverers, which resumed growth before the experiment ended, and arresters, which remain arrested. We calculated the average lag duration of the recoverers and their corresponding fraction ϕ within each population. We observed a robust inverse relationship where populations with longer lags had smaller fractions of recoverers (Fig. 5c). This empirical trend matches well with predictions from our two-phenotype model.

## IV. DISCUSSION

Microbial populations repeatedly exposed to environmental shifts must reconcile fast growth in the current niche with the capacity to resume growth after a switch. In this study, we investigated the role of phenotypic heterogeneity in microbial populations, focusing on the adaptation of yeast to changing carbon sources. The central experimental observation motivating this work is that following a switch from a glucose-rich environment to a maltose-rich environment (or other environments), a substantial fraction of genetically identical yeast cells remain arrested in lag phase for the entire duration of the experiment [14–17]. This persistent growth arrest suggests that cells in this subpopulation occupy a distinct physiological state, raising the question whether this heterogeneity might reflect an evolved strategy to optimize population-level fitness in fluctuating environments. To address this, we developed a general framework based on dynamic programming to determine the optimal bet-hedging strategies for microbial populations transitioning between environments where cells can vs. cannot readily reorganize gene expression. We found that the optimal phenotype distribution consists of at most two discrete phenotypes, even when a continuous range of phenotypes is allowed. Under certain conditions, the population adopts a bet-hedging strategy composed of two distinct specialists: one phenotype minimizes lag to recover quickly in the non-preferred environment (NP), while the other maximizes growth in the preferred environment (P) but never recovers in NP. In other regimes, a single optimal generalist phenotype emerges that balances growth and lag.

A key prediction from our model is a nontrivial relationship between the fraction of recoverers and their lag. This relationship is a consequence of imposing optimality of the phenotype distribution for a given physiological trade-off across different environmental transition rates. This prediction matches single-cell datasets spanning wild strains, evolved lines, and varying pre-growth nutrient durations, suggesting an underlying constraint in the genetic circuit that mediates phenotypic diversification.

Two assumptions underpin our results that populations benefit from having phenotypic heterogeneity with distinct discrete phenotypes: First, the population can re-tune its phenotype distribution rapidly in the preferred environment (P) but phenotypes are locked during lag in the non-preferred environment (NP). Second, a monotonic trade-off links higher pre-shift growth to longer post-shift lag. Other ingredients of the model, such as exponentially distributed dwell times, the exact form of the trade-off, and the perfect arrester limit (ℓ→∞), allow analytic tractability but are not required for heterogeneity to be favored. More complex dwell-time distributions could create regimes where more than two distinct specialists make up the optimal strategy, each tuned to a different characteristic timescale of environmental shifts.

Mechanistically, a minimal metabolic model motivates why a trade-off may exist: allocating enzymes to drive flux in the pre-shift direction (fast growth) delays net flux reversal and consequently delays resumed growth after the switch. Systematic variation of pre-shift enzyme allocation reproduces a power-law growth-lag trade-off curve consistent with the phenomenology encapsulated by Eq. 7.

Our framework extends classical bet-hedging theory [3, 6, 7, 12] in a key way: We couple pre-shift growth and post-shift lag through an explicit metabolic constraint that encodes how proteome allocation before the switch creates a bottleneck in the speed of adaptation after the switch. This makes the trade-off mechanistic rather than just phenomenological. Our work is thus novel in that we link cellular physiology to population level growth outcomes in fluctuating conditions.

Lag heterogeneity following carbon shifts in *S. cerevisiae* shares a key similarity with persister formation in *E. coli*, competence in *Bacillus subtilis*, and sporulation in filamentous fungi: all involve a slow-growing subpopulation that sacrifices immediate proliferation for future advantage. In these systems, phenotypic diversity provides a benefit to the population by being prepared for unfavorable conditions. The “recoverer” state in our model plays this protective role, maintaining the population (e.g. against competitors) until favorable conditions return.

Our analysis suggests that the recoverer lag-fraction relationship can function as an evolutionary “rheostat”, providing populations with a mechanism to tune their balance between fast growth and lag. In our metabolic model, such a rheostat could be wired through regulatory control of proteome allocation. Changes in the expression of enzymes that bias flux toward pre-shift growth would continuously shift both the growth rate in the preferred environment and the lag after a nutrient switch. This would enable populations to smoothly adjust their phenotypic distribution in response to the statistics of environmental change, without requiring rewiring of their metabolic circuitry. More broadly any modulation (genetic or otherwise) of upstream regulatory pathways that control the phenotype distribution will then satisfy an optimality constraint that can be tuned to the rate of environmental change, offering a convenient evolutionary rheostat for rapidly adapting to new environments. In short, with a single control knob, closely related strains constrained by the same trade-off could flexibly adapt to different environmental fluctuation statistics.

Promising directions for future experimental work include (i) varying ω and $\omega^{\prime }$ (e.g. in a microfluidic setup) to test the predicted re-entrant generalist–specialist–generalist behavior, (ii) adjusting pre-shift enzyme allocation (genetically or via growth history) to test the coupling between growth and lag, and (iii) measuring the survival of long-lag arresters to test for a “perfect arrester” state. Together, these experiments would show whether the recoverer fraction–lag relation functions as an evolutionary rheostat across strains.

## Supplementary Material

1

## Figures and Tables

**TABLE I.** Values for parameters used in the metabolic model.

Parameters	Values
$G_{\text{P}},N_{\text{P}}$	2.0, 0.0 a.u.
$G_{\text{NP}},N_{\text{NP}}$	0.0, 1.0 a.u.
r,δ	1.0, 3.0 a.u. of 1/time
$K_{g},K_{n},K_{\text{BM}}$	2.0, 2.0, 1.0 a.u.
ν	2

## Data Availability

The data and code used to generate all figures are available at https://doi.org/10.5281/zenodo.17582890.
