# Supplementary material for: Phenotypic heterogeneity in lag reflects an evolutionarily stable bet-hedging strategy
Source: bioRxiv. 2025 Nov 11:2025.11.02.686100. Preprint. [Version 2] doi: 10.1101/2025.11.02.686100 (PMC12642563; doi:10.1101/2025.11.02.686100)
Supplement: 1 [file NIHPP2025.11.02.686100V2-supplement-1.pdf]

bioRxiv preprint doi: https://doi.org/10.1101/2025.11.02.686100; this version posted November 11, 2025. The copyright holder for this preprint (which was not certified by peer review) is the author/funder, who has granted bioRxiv a license to display the preprint in perpetuity. It is made available under aCC-BY 4.0 International license.

Appendix A: Experimental data

Data for single-cell lag phase measurements was taken from New et al. 2014 and Cerulus et al. 2018. In short, cells were pre-grown in glucose, harvested, and transferred to media containing maltose. The lag duration of individual cells was measured using automated time-lapse microscopy, defined as the time from transfer until the first observable sign of division (bud emergence or resumption of bud growth). For experimental details, refer to New et al. 2014. To generate the distribution of single-cell lag durations (Fig. 1b), data was specifically taken from Dataset S1. It contains single-cell lag phase measurements for 18 strains of *S. cerevisiae* and we picked the distribution for strain 'DBVPG1373' for Fig. 1b in the main text. Figure S1 contains the lag distributions of other strains from their dataset which highlights a bimodality in lag durations. For Fig. 5b in the main text, we took single-cell lag data for wild yeast strains and evolved strains from Dataset S1 and Dataset S2 of New et al. 2014 (Fig. S2a,b), respectively, and for varying pre-shift durations in glucose from Cerulus et al. 2018 (Fig. S2c), to estimate the lag and fraction of recoverers in a population. Raw single-cell data from [15] presented in Fig. S2c were shared through personal communication by Bram Cerulus and Kevin Verstrepen.

Most single-cell lag distributions are bimodal, i.e., composed of a group of recoverer cells that recover from lag and arrester cells which do not resume growth until the end of the experiment. We compute the mean lag duration of the recoverer population for each strain and plot it against their fraction in the population.

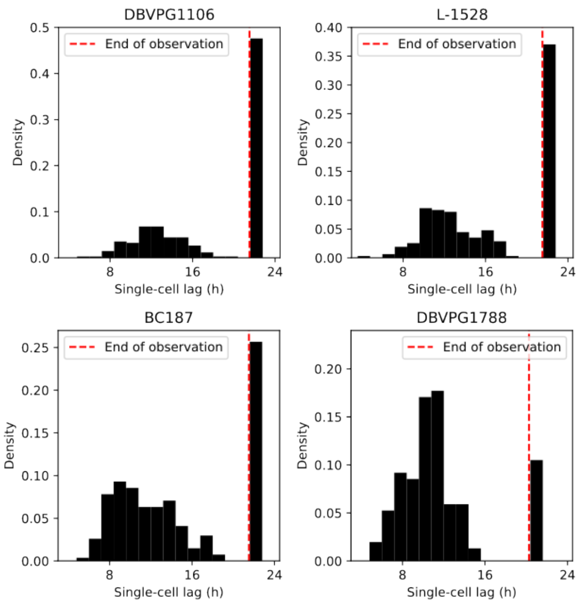

Fig. S1. Single-cell lag distributions of different yeast strains.

Appendix B: Cellular decision-making framework

1. Phenotype heterogeneity in fluctuating environments

We consider a scenario where a cellular population experiences transitions between a preferred environment (P) and a non-preferred environment (NP). Cells can take any one of K possible phenotypes. For example, a cell with a phenotype that allows for rapid growth in P may experience a longer lag phase or greater death rate when the environment switches to NP. In P, cells grow and rapidly switch their phenotypes so as to maintain a phenotype distribution, $\boldsymbol{\phi} = (\phi_1, \phi_2, ..., \phi_K)$ (with $\sum_k \phi_k = 1$), within the population. The selection of a phenotype distribution could occur through stochastic switching between phenotype states; we discuss this further in Section F. The distinction between P and NP is that cells *cannot* choose their phenotype distribution in NP. This corresponds to the case where cells experience a non-growing phase (lag, starvation, or death) in NP. In this non-growing phase, the population cannot readily express genes and synthesize new proteins, and thus cannot arbitrarily choose its phenotype distribution.

The state of the environment is a stochastic process. The instantaneous rate at which the population switches from P to NP (denoted ω) or from NP to P (denoted ω') could have arbitrary dependence on the history of environmental states (for example, the time since the last switch from NP to P). Cells in P can *anticipate* the transition from P to NP and choose their phenotype distribution $\boldsymbol{\phi}$ so as to maximize the long-term growth rate of the population. In the main text, we present a simplified version where cells make decisions that are independent

bioRxiv preprint doi: https://doi.org/10.1101/2025.11.02.686100; this version posted November 11, 2025. The copyright holder for this preprint (which was not certified by peer review) is the author/funder, who has granted bioRxiv a license to display the preprint in perpetuity. It is made available under aCC-BY 4.0 International license.

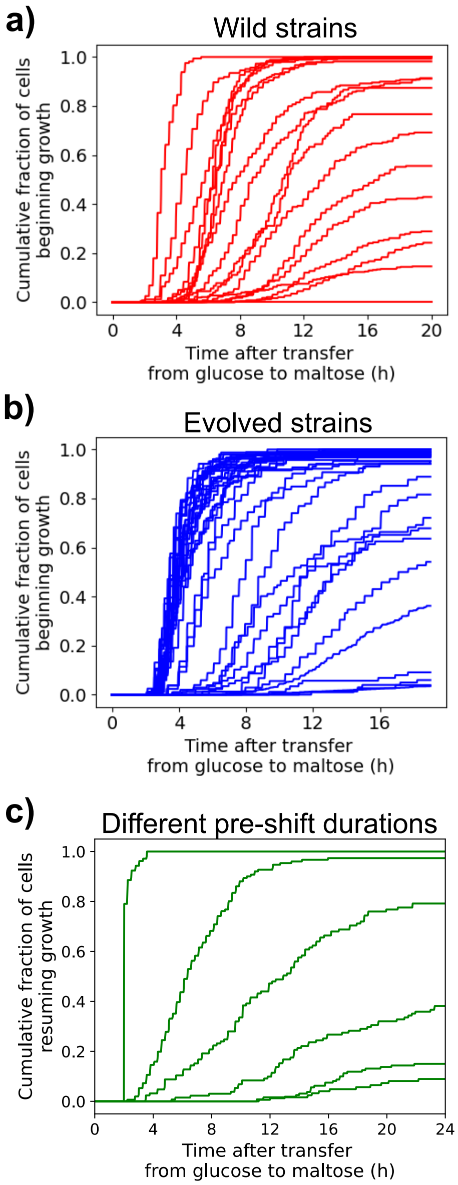

Fig. S2. (a) Cumulative fraction of cells resuming growth after the nutrient switch for wild strains of yeast. (b) Cumulative fraction of cells resuming growth after the nutrient switch for evolved populations of yeast. (c) Cumulative fraction of cells resuming growth after the nutrient switch for populations experiencing varying pre-shift durations in glucose.

of history. In this simplified version, the environment switches from P to NP and NP to P at constant rates ω and ω' respectively. Here, we present a general version of the framework where cellular decisions are allowed to be history-dependent.

2. Model setup

We developed a general optimization framework based on dynamic programming to investigate how phenotype heterogeneity influences population fitness under the scenario described in the previous section. This framework allows us to model cellular decision-making using methods similar to those used in reinforcement learning and control theory.

Let $s(t)$ represent the state at time t and $\gamma(s(t), k)$ denote the instantaneous growth rate of a cell with phenotype k in state $s(t)$. $s(t)$ includes information about whether the current environment is P or NP, and possibly additional information about the growth medium in P (which will affect instantaneous growth rate), the time since the most recent shift from P to NP (which is important to incorporate lag), or historical information the cell uses to better anticipate future environmental transitions. The probability of transitioning from state s to s' in interval dt is given by the transition probability $P(s(t+dt) = s'|s(t) = s)$. This transition probability is time-translation invariant, i.e., there is no dependence on absolute time except through s. Note that the framework applies for a general state space $s(t)$, and it can thus capture potentially complex historical dependencies.

Given $s(t)$ and the phenotype distribution $\boldsymbol{\phi}(t)$, the instantaneous growth rate of a population of N cells is

$$\frac{1}{N}\frac{dN}{dt} = \sum_{k=1}^{K} \phi_k(t)\gamma(s(t), k) \equiv \bar{\gamma}(s(t), \boldsymbol{\phi}(t)). \tag{B1}$$

If the initial number of cells is N_0 and the number of cells after time T is N_T, the long-term growth rate is

$$\Gamma_{\mathrm{T}} \equiv \frac{1}{T}\log\frac{N_T}{N_0} = \frac{1}{T}\int_0^T \langle\bar{\gamma}(s(t), \boldsymbol{\phi}(t))\rangle dt, \tag{B2}$$

where the angled brackets denote an expectation over changes in environmental state. Our goal is to find the optimal strategy to select $\boldsymbol{\phi}$ in P such that Γ_T is maximized as $T \to \infty$.

Instead of directly maximizing $T^{-1}\int_0^T \langle\bar{\gamma}(s(t), \boldsymbol{\phi}(t))\rangle dt$ as $T \to \infty$, we find the strategy that maximizes $\int_0^\infty e^{-\mu t}\langle\bar{\gamma}(s(t), \boldsymbol{\phi}(t))\rangle dt$ as $\mu \to 0$. This alternative objective is commonly used to remove time as a state variable when computing the optimal strategy. No singularities arise in the limit $\mu \to 0$ as the decision to pick a particular $\boldsymbol{\phi}$ matters only until the next time the population encounters P.

A population in P selects its phenotype distribution $\boldsymbol{\phi}$ at every interval dt (where $1/dt$ is much larger than the typical growth rate of the cells in P). We now derive our central optimization objective. We write down a (Bellman) dynamic programming equation for the long-term growth rate *given* that the population is in state s in environment P, assuming the population makes the best possible choice for the phenotype distribution $\boldsymbol{\phi}$. Let's call this optimal long-term growth rate $\Gamma_{\mathrm{P}}(s)$. We have

$$\Gamma_{\mathrm{P}}(s) = \max_{\boldsymbol{\phi}}\left\{\int_0^\infty dt e^{-\mu t}\langle\bar{\gamma}(s(t), \boldsymbol{\phi}(t))\rangle_{s(0)=s,\boldsymbol{\phi}(0)=\boldsymbol{\phi}}\right\}, \tag{B3}$$

bioRxiv preprint doi: https://doi.org/10.1101/2025.11.02.686100; this version posted November 11, 2025. The copyright holder for this preprint (which was not certified by peer review) is the author/funder, who has granted bioRxiv a license to display the preprint in perpetuity. It is made available under aCC-BY 4.0 International license.

where the expectation (represented by the angled brackets) is over possible future states given the current state $s(0) = s$ and the phenotype distribution $\boldsymbol{\phi}$. Splitting the integral,

$$\Gamma_{\rm P}(s) = \max_{\boldsymbol{\phi}} \Bigg\{ \overline{\gamma}_{\rm P}(s,\boldsymbol{\phi})dt + \int_{dt}^{\infty} dt e^{-\mu t} \langle \overline{\gamma}(s(t),\boldsymbol{\phi}(t))\rangle_{s(dt),\boldsymbol{\phi}(dt)|s(0)=s,\boldsymbol{\phi}(0)=\boldsymbol{\phi}} \Bigg\}, \tag{B4}$$

where the expectation is now over the state of the population at dt given that at $t = 0$ it is at $s(0) = s$, $\boldsymbol{\phi}(0) = \boldsymbol{\phi}$. Setting $t = t' + dt$, we have

$$\Gamma_{\rm P}(s) = \max_{\boldsymbol{\phi}} \Bigg\{ \overline{\gamma}_{\rm P}(s,\boldsymbol{\phi})dt + e^{-\mu dt} \int_{0}^{\infty} dt' e^{-\mu t'} \langle \overline{\gamma}(s(t'),\boldsymbol{\phi}(t'))\rangle_{s(0),\boldsymbol{\phi}(0)|s(-dt)=s,\boldsymbol{\phi}(-dt)=\boldsymbol{\phi}} \Bigg\}, \tag{B5}$$

where we used the fact that the transition matrix $P(s(t+dt),\boldsymbol{\phi}(t+dt)|s(t),\boldsymbol{\phi}(t))$ is time-translation invariant.

Given that the population is in P at state s, there are two possibilities: after dt, either the environment stays in P with probability $1-\omega(s)dt$, or switches to NP with probability $\omega(s)dt$. If the environment stays in P and the state transitions to s', then the expected long-term growth rate after dt is $\Gamma_{\rm P}(s')$. If the environment transitions to NP and the state transitions to s', we will denote the expected long-term growth rate by $\Gamma_{\rm NP}(s',\boldsymbol{\phi})$ (which we specify further below). We split the integral in the above equation based on the probability of these two possibilities,

$$\Gamma_{\rm P}(s) = \max_{\boldsymbol{\phi}} \Bigg\{ \overline{\gamma}_{\rm P}(s,\boldsymbol{\phi})dt + (1-(\omega(s)+\mu)dt)\langle\Gamma_{\rm P}(s')\rangle + \omega(s)dt\langle\Gamma_{\rm NP}(s',\boldsymbol{\phi})\rangle \Bigg\}, \tag{B6}$$

where we have dropped the subscripts specifying that the expectations are over the state and phenotype distribution at the subsequent time step, i.e., $s', \boldsymbol{\phi}'$, given s at the current time step. Importantly, the choice of $\boldsymbol{\phi}$ does not matter if the environment stays in P. Moreover, the term involving $\langle\Gamma_{\rm P}(s')\rangle$ can be ignored when computing the optimal strategy. To obtain the optimal strategy $\boldsymbol{\phi}^*$, we simply have

$$\boldsymbol{\phi}^*(s) = \arg\max_{\boldsymbol{\phi}} \Big\{ \overline{\gamma}(s,\boldsymbol{\phi}) + \omega(s)\langle\Gamma_{\rm NP}(s',\boldsymbol{\phi})\rangle \Big\} \tag{B7}$$

Now, we unpack $\Gamma_{\rm NP}(s',\boldsymbol{\phi})$. Let $\Pi(\tau)$ be the probability density that the environment stays in NP for duration τ and let s'' be the state that the environment ends up in after the switch back to P after interval τ. We have

$$\Gamma_{\rm NP}(s',\boldsymbol{\phi}) = \int_0^{\infty} d\tau\, \Pi(\tau) \bigg(\int_0^{\tau} dt\, e^{-\mu t} \langle \overline{\gamma}_{\rm NP}(s(t),\boldsymbol{\phi}(t))\rangle_{s(t)} + e^{-\mu\tau}\langle\Gamma_{\rm P}(s'')\rangle_{s''} \bigg) \tag{B8}$$

where $s(t)$ and $\boldsymbol{\phi}(t)$ are now the state and distribution of phenotypes at time t after the switch to NP given that the state and distribution were s' and $\boldsymbol{\phi}$ when the switch occurred. The first term on the right hand side does depend on μ, but it does not contribute to the calculation of $\boldsymbol{\phi}^*$ if $\Pi(\tau)$ decays to zero on timescales much smaller than $1/\mu$. The second term on the right hand side contains $\langle\Gamma_{\rm P}(s'')\rangle$ which is the expected long-term growth rate once the environment switches back to P. This term does not depend on $\boldsymbol{\phi}$ and can therefore be ignored when finding the optimal $\boldsymbol{\phi}$. We arrive at

$$\boldsymbol{\phi}^*(s) = \arg\max_{\boldsymbol{\phi}} \Bigg\{ \overline{\gamma}_{\rm P}(s,\boldsymbol{\phi}) + \omega(s) \left\langle \int_0^{\infty} d\tau\, \Pi(\tau) \left(\int_0^{\tau} dt\, \overline{\gamma}_{\rm NP}(s(t),\boldsymbol{\phi}(t)) \right) \right\rangle_{s(t)} \Bigg\}. \tag{B9}$$

Interchanging integrals and defining $F(t) = \int_t^{\infty} d\tau \Pi(\tau)$, we re-write the above as

$$\boldsymbol{\phi}^*(s) = \arg\max_{\boldsymbol{\phi}} \Bigg\{ \overline{\gamma}_{\rm P}(s,\boldsymbol{\phi}) + \omega(s) \left\langle \int_0^{\infty} dt F(t) \overline{\gamma}_{\rm NP}(s(t),\boldsymbol{\phi}(t)) \right\rangle_{s(t)} \Bigg\}. \tag{B10}$$

The average growth rate after time t in NP is

$$\overline{\gamma}_{\rm NP}(s(t),\boldsymbol{\phi}(t)) = \frac{\sum_{k=1}^{K} \phi_k \gamma_{\rm NP}(s(t'),k) e^{\int_0^t dt' \gamma_{\rm NP}(s(t'),k)}}{\sum_{k'=1}^{K} \phi_{k'} e^{\int_0^t dt'' \gamma_{\rm NP}(s(t'),k')}}, \tag{B11}$$

$$= \frac{d}{dt} \log \left(\sum_{k=1}^{K} \phi_k e^{\int_0^t dt' \gamma_{\rm NP}(s(t'),k)} \right). \tag{B12}$$

Plugging this expression into Eq. B10 and integrating by parts, we finally obtain the objective

$$\boldsymbol{\phi}^*(s) = \arg\max_{\boldsymbol{\phi}} \Bigg\{ \overline{\gamma}_{\rm P}(s,\boldsymbol{\phi}) + \omega(s) \left\langle \int_0^{\infty} d\tau\, \Pi(\tau) \log \left(\sum_{k=1}^{K} \phi_k e^{\int_0^{\tau} dt \gamma_{\rm NP}(s(t),k)} \right) \right\rangle_{s(t)} \Bigg\}$$

$$\text{subject to } \sum_k \phi_k = 1 \text{ and } \phi_k \geq 0 \text{ for all } k. \tag{B13}$$

bioRxiv preprint doi: https://doi.org/10.1101/2025.11.02.686100; this version posted November 11, 2025. The copyright holder for this preprint (which was not certified by peer review) is the author/funder, who has granted bioRxiv a license to display the preprint in perpetuity. It is made available under aCC-BY 4.0 International license.

This expression has an intuitive interpretation: the optimal strategy balances the instantaneous growth rate in P and the expected long-term growth rate if the environment were to transition to NP. We now show how this framework can be used to find the optimal decision-making strategy in two scenarios involving a growth-lag trade-off and a growth-death trade-off.

3. Balancing growth and lag

Equation B13 is applicable generally when cells transition between preferred and non-preferred environments. In this section, we discuss the situation where a population of cells experiences rapid growth in the preferred environment (P) and undergoes lag phase before resuming growth in the non-preferred environment (NP). In P, cells grow such that $\gamma(\mathrm{P},k) = \gamma_k$. In NP, cells experience lag before they can resume growth: $\gamma(\tau_{\mathrm{NP}},k) = \gamma'\Theta(\tau_{\mathrm{NP}} - \ell_k)$, where τ_{NP} is the time spent in NP since the last switch from P and ℓ_k is the lag of phenotype k. We consider $\Pi(\tau) = \omega' e^{-\omega' t}$ and also ignore any other dependence on the state s. Interchanging the integrals over τ and t in Eq. B13, we get

$$\boldsymbol{\phi}^* = \arg\max_{\boldsymbol{\phi}} \left\{ \sum_{i=1}^{K} \phi_i \gamma_i + \omega \int_0^\infty dt e^{-\omega' t} \log\left[\sum_{i=1}^{K} \phi_i e^{\gamma'[t-\ell_i]_+} \right] \right\}. \tag{B14}$$

Making substitutions: $t' = \gamma' t$, $\ell_i' = \ell_i \gamma'$, $\tilde{\gamma}_i = \gamma_i/\omega$, and $\eta = \gamma'/\omega'$, we now have

$$\boldsymbol{\phi}^* = \arg\max_{\boldsymbol{\phi}} \left\{ \sum_{i=1}^{K} \phi_i \tilde{\gamma}_i + \int_0^\infty dt' e^{-\frac{t'}{\eta}} \log\left[\sum_{i=1}^{K} \phi_i e^{[t'-\ell_i']_+} \right] \right\}. \tag{B15}$$

4. Balancing growth and death

In an alternative paradigm, rather than experiencing a lag phase in the non-preferred environment (NP), cells are subjected to lethal conditions and die at phenotype-specific rates. In this framework, phenotype variation is characterized not by differences in lag durations, but by distinct death rates ξ_k associated with each phenotype in NP. In P, cells grow such that $\gamma(\mathrm{P},k) = \gamma_k$ and in NP, cells die such that $\gamma(\mathrm{NP},k) = \xi_k$, where $\xi_k (\leq 0)$ is the death rate for phenotype k. Similar to the previous section, we use Eq. B13 and modify the second term concerned with long-term growth in NP,

$$\boldsymbol{\phi}^* = \arg\max_{\boldsymbol{\phi}} \left\{ \sum_{i=1}^{K} \phi_i \gamma_i + \omega \int_0^\infty dt e^{-\omega' t} \log\left[\sum_{i=1}^{K} \phi_i e^{\xi_i t} \right] \right\}. \tag{B16}$$

After making substitutions: $t' = \omega' t$, $\overline{\xi_i} = \xi_i/\omega'$ and $\tilde{\gamma}_i = \gamma_i/\omega$, we have

$$\boldsymbol{\phi}^* = \arg\max_{\boldsymbol{\phi}} \left\{ \sum_{i=1}^{K} \phi_i \tilde{\gamma}_i + \int_0^\infty dt' e^{-t'} \log\left[\sum_{i=1}^{K} \phi_i e^{\overline{\xi_i} t'} \right] \right\}. \tag{B17}$$

We determine the optimal phenotype distribution $\boldsymbol{\phi}^*$ numerically.

5. Numerical optimization

We assume the population contains a distribution over K phenotypes, where phenotype k (where k takes on values from 1 to K) has growth rate γ_k ($\gamma_{\min} \leq \gamma_k \leq \gamma_{\max}$) in P. In the growth-lag scenario, phenotypes are characterized with lags ℓ_k in NP and all phenotypes have the same growth rate γ' in NP when cells recover from lag phase. In the growth-death scenario, phenotypes are characterized with death rates ξ_k in NP. The phenotype distribution is given by $\boldsymbol{\phi} = (\phi_1, .., \phi_k, .., \phi_K)$.

Step-by-step procedure to run the optimization scheme:

- First we pick the parametric form for the trade-off between growth and lag (or death). This gives us growth rates $\{\gamma_i\}$ in P and the corresponding lag durations $\{\ell_i\}$ (or death rates $\{\xi_i\}$) in NP.
- Choose transition rates ω and ω'.
- Initialize $\boldsymbol{\phi} = \{\phi_i\}$ such that $\phi_i = 1/K$. To prevent having to enforce an extra constraint of $\sum_{i=1}^{K} \phi_i = 1$ during optimization, we parametrize the probabilities as $\phi_i = \frac{e^{\beta_i}}{1+\sum_{k=2}^{K} e^{\beta_k}}$ for $2 \leq i \leq K$ and $\phi_1 = \frac{1}{1+\sum_{k=2}^{K} e^{\beta_k}}$. Here $\{\beta_i\}$ are unconstrained parameters that we can directly optimize over.
- Using the Adam optimizer in the Optax library applied to a JAX implementation in Python, we numerically find $\{\beta_i\}$ that maximizes $\Gamma_{\mathrm{P}}(\boldsymbol{\phi})$ from Eq. B15. We then obtain the optimal phenotype distribution $\boldsymbol{\phi}^*$.

Appendix C: Optimal phenotype distribution consists of a few discrete phenotypes

We consider our optimization problem where the population selects a phenotype distribution $\boldsymbol{\phi}$ to maximize a long-term growth objective of the general form

$$\Gamma(\boldsymbol{\phi}) = \max_{\boldsymbol{\phi}} \left\{ \sum_{i=1}^{K} \phi_i \tilde{\gamma}_i + \int_0^\infty dt' e^{-\frac{t'}{\eta}} \log\left[\sum_{i=1}^{K} \phi_i e^{[(t'-\ell_i']_+} \right] \right\}, \tag{C1}$$

with the constraints:

- $\phi_i \geq 0 \quad \forall i$

bioRxiv preprint doi: https://doi.org/10.1101/2025.11.02.686100; this version posted November 11, 2025. The copyright holder for this preprint (which was not certified by peer review) is the author/funder, who has granted bioRxiv a license to display the preprint in perpetuity. It is made available under aCC-BY 4.0 International license.

- $\sum_{i=1}^{K} \phi_i = 1$.

For this constrained nonlinear optimization problem, we use the Karush-Kuhn-Tucker (KKT) conditions to make predictions about the structure of the optimal solution. We construct the corresponding Lagrangian function,

$$\mathcal{L}(\boldsymbol{\phi}, \lambda, \mu) = -\Gamma(\boldsymbol{\phi}) + \sum_{i=1}^{K} \mu_i \phi_i + \lambda \left(\sum_{i=1}^{K} \phi_i - 1 \right). \quad \text{(C2)}$$

for some Lagrange multiplier λ and KKT multipliers $\mu_i \geq 0$. To maximize $\Gamma(\boldsymbol{\phi})$ given the constraints, the stationarity condition requires

$$-\frac{\partial \mathcal{L}}{\partial \phi_k} = \frac{\partial \Gamma}{\partial \phi_k} - \mu_k - \lambda = 0. \quad \text{(C3)}$$

Let $g_k(\boldsymbol{\phi}) = \frac{\partial \Gamma(\boldsymbol{\phi})}{\partial \phi_k}$ denote the marginal value of phenotype k. Then the support of the optimal solution (i.e., the set of phenotypes with $\phi_k > 0$) is determined by the set of indices where $g_k = \lambda$. We get this from the complementary slackness condition which requires that $\mu_i \phi_i = 0$ for $i = 1, 2, ..., K$. Geometrically, this corresponds to the intersection points of a horizontal line at height λ with the graph of g_k as a function of phenotype lag ℓ'_k. Let's examine the explicit form of g_k:

$$g_k(\boldsymbol{\phi}) = \frac{\partial \Gamma(\boldsymbol{\phi})}{\partial \phi_k} = \tilde{\gamma}_k + \int_0^\infty dt' \Pi(t') \frac{e^{[t' - \ell'_k]_+}}{\sum_{i=1}^{K} \phi_i e^{[t' - \ell'_i]_+}}. \quad \text{(C4)}$$

In our framework, the rescaled lag durations ℓ' and preferred-environment growth rates $\tilde{\gamma}$ are strictly ordered such that: $\ell'_1 < \ell'_2 < \cdots < \ell'_K$ and $\tilde{\gamma}_1 < \tilde{\gamma}_2 < \cdots < \tilde{\gamma}_K$. This implies that the first term in $g_k(\boldsymbol{\phi})$ monotonically increases with k. In the second term, the dwell time distribution $\Pi(t')$ acts as a discount factor such that for larger t', $\Pi(t')$ is smaller since the distribution monotonically decreases with t'. This implies that the contribution to the integral for larger ℓ'_k is reduced which in turn means that the second term in $g_k(\boldsymbol{\phi})$ monotonically decreases with k. $g_k(\boldsymbol{\phi})$ is composed of the sum of two terms with opposing monotonicities. If g_k is strictly monotonic in ℓ'_k, then the horizontal line intersects the curve at most once, implying that the optimal strategy is supported on a single phenotype. The number of times g_k intersects with the horizontal line determines the number of phenotypes with non-zero probability. For well-behaved $\Pi(t)$, we expect at most a few discrete phenotypes, though the precise number depends on $\Pi(t)$ and the growth-lag trade-off.

Single timescale in NP

In this section, we present a geometric argument for why we expect at most two phenotypes when the distribution of dwell times in NP is unimodal. This geometric argument complements the formal argument in the main text using the KKT theorem. Suppose that the time spent in NP is a constant t instead of being exponentially distributed. We have for K phenotypes (with arbitrary growth rates)

$$\Gamma(\boldsymbol{\phi}) = \sum_k \gamma_k^{\mathrm{P}} \phi_k + \log \left(\sum_k \phi_k e^{\gamma_k^{\mathrm{NP}}(t)} \right), \quad \text{(C5)}$$

where γ_k^{P} is the growth rate in P and $\gamma_k^{\mathrm{NP}}(t)$ is the effective growth rate in NP for phenotype k. In the growth-lag scenario $\gamma_k^{\mathrm{NP}}(t) = \gamma'[t - \ell_k]_+$, while in the growth-death scenario $\gamma_k^{\mathrm{NP}}(t) = \xi_k t$.

Consider $\boldsymbol{\phi}$ such that $\sum_k \phi_k \gamma_k^{\mathrm{P}} = y$ and $\sum_k \phi_k e^{\gamma_k^{\mathrm{NP}}(t)} = x$. The two constraints $\phi_k \geq 0$ and $\sum_k \phi_k = 1$ define a closed feasible region in $\boldsymbol{\phi}$. This closed feasible region in $\boldsymbol{\phi}$ translates to a closed feasible region D in the two-dimensional (x, y) plane. Consider the family of curves $y = \Gamma - \log x$ parameterized by Γ. As Γ is decreased from infinity, the optimal feasible solution is due to the curve that just touches D. Now we show that the boundary points of D correspond to ϕ such that at most two of the phenotypes have non-zero probability mass.

First note that the intersection of the hyperplane $\sum_k \phi_k e^{\gamma_k^{\mathrm{NP}} t} = x$ with the probability simplex $\phi_k \geq 0$, $\sum_k \phi_k = 1$ is a convex hull formed by points on line segments joining two vertices of the probability simplex. These are points that have non-zero probability for at most two of the phenotypes. Next, for a given value of x, the top and bottom boundaries of D correspond to the maximum and minimum value of y taken over this convex hull. However, the maximum and minimum of y is always achieved at the vertices of the convex hull. The optimal solution therefore is such that at most two of the phenotypes have non-zero mass.

While this argument does not generalize to arbitrary distributions over t, it helps rationalize why at most two phenotypes are largely sufficient when the distribution over t has one typical timescale (such as for an exponential distribution).

Appendix D: Under what scenarios is a bimodal solution favored?

1. Growth–lag paradigm

We aim to obtain a condition on the curvature of the growth-lag trade-off relation $\gamma(\ell)$ for when the one-state solution splits into a two-state solution. We first obtain the best one-state solution and then ask if there is a better two-state solution such that the two states have lags that are in an ε-neighborhood of the best single-state solution.

bioRxiv preprint doi: https://doi.org/10.1101/2025.11.02.686100; this version posted November 11, 2025. The copyright holder for this preprint (which was not certified by peer review) is the author/funder, who has granted bioRxiv a license to display the preprint in perpetuity. It is made available under aCC-BY 4.0 International license.

Consider the general two-state objective function

$$\Gamma(\phi,\ell_1,\ell_2) = (\phi\gamma_{\rm P}(\ell_1) + (1-\phi)\gamma_{\rm P}(\ell_2)) \\ +\omega\int_0^\infty dt\Pi(t)\log\left(\phi e^{[\gamma'(t-\ell_1)]_+} + (1-\phi)e^{[\gamma'(t-\ell_2)]_+}\right). \tag{D1}$$

We set $\omega = \gamma' = 1$ noting that growth rates in P are measured in units of ω and time spent in NP and lag durations are measured in units of $1/\gamma'$. The optimal single-state solution (with $\phi = 1$ in Eq. D1 denoted by Γ_1 is obtained by maximizing

$$\Gamma_1 = \max_\ell\left\{\gamma_{\rm P}(\ell) + \int_0^\infty dt\Pi(t)(t-\ell)\right\}, \tag{D2}$$

which leads to the optimality condition and single-state growth rate

$$\left.\frac{d\gamma_{\rm P}}{d\ell}\right|_{\ell_0} = F(\ell_0), \quad \Gamma_1 = \gamma_{\rm P}(\ell_0) + I(\ell_0), \tag{D3}$$

where ℓ_0 is the lag that maximizes the rhs of Eq. D2, and we denote $F(\ell) = \int_\ell^\infty dt\Pi(t)$ and $I(\ell) = \int_0^\infty dt\Pi(t)(t-\ell) = \int_\ell^\infty dtF(t)$ (the latter is obtained via integration by parts). We only consider cases where the best single-state lag ℓ_0 is finite, that is, $\Gamma_1 > \gamma_{\rm P}(\infty)$.

We now calculate the best two-state solution such that $\ell_0-\varepsilon \le \ell_1,\ell_2 \le \ell_0+\varepsilon$ where ε is a small positive constant. To do this, we expand each term in Eq. D1 to second order. Define $\varepsilon_1 = \ell_1-\ell_0$, $\varepsilon_2 = \ell_2-\ell_0$. All γ terms and its derivatives are evaluated at ℓ_0. The first term on the rhs is

$$\phi\gamma_{\rm P}(\ell_1) + (1-\phi)\gamma_{\rm P}(\ell_2) = \gamma + \frac{d\gamma_{\rm P}}{d\ell}(\varepsilon_1\phi + \varepsilon_2(1-\phi)) \\ + \frac{1}{2}\frac{d^2\gamma_{\rm P}}{d\ell^2}(\varepsilon_1^2\phi + \varepsilon_2^2(1-\phi)). \tag{D4}$$

We split the second term to three parts

$$\int_0^\infty dt\Pi(t)\log\left(\phi e^{t-\ell_1} + (1-\phi)e^{t-\ell_2}\right) \\ = \int_{\ell_1}^{\ell_2} dt\Pi(t)\log(\phi e^{t-\ell_1} + (1-\phi)) + \\ \int_{\ell_2}^\infty dt\Pi(t)(t-\ell_2) + \int_{\ell_2}^\infty dt\Pi(t)\log(\phi e^{\ell_2-\ell_1} + (1-\phi)). \tag{D5}$$

The first term in Eq. D5 to second order in ε is

$$\int_{\ell_1}^{\ell_2} dt\Pi(t)\log(\phi e^{t-\ell_1} + (1-\phi)) \approx \frac{1}{2}\phi\Pi(\ell_0)(\varepsilon_2-\varepsilon_1)^2. \tag{D6}$$

The second term in Eq. D5 to second order in ε is

$$\int_{\ell_2}^\infty dt\Pi(t)(t-\ell_2) = \int_{\ell_2}^\infty dtF(t) \approx I(\ell_0) \\ - \varepsilon_2F(\ell_0) + \frac{1}{2}\varepsilon_2^2\Pi(\ell_0). \tag{D7}$$

The third term in Eq. D5 to second order in ε is

$$\int_{\ell_2}^\infty dt\Pi(t)\log(\phi e^{\ell_2-\ell_1} + (1-\phi)) \approx (\varepsilon_2-\varepsilon_1)\phi F(\ell_0) \\ - \varepsilon_2(\varepsilon_2-\varepsilon_1)\phi\Pi(\ell_0) + \frac{1}{2}(\varepsilon_2-\varepsilon_1)^2\phi(1-\phi)F(\ell_0). \tag{D8}$$

Collecting the zeroth order terms, we get $\gamma_{\rm P}(\ell_0) + I(\ell_0)$, which is precisely Γ_1. The first order terms cancel out exactly once we use the stationarity condition $d\gamma_{\rm P}/d\ell = F(\ell_0)$ at ℓ_0. After adding up the contributions from the second order terms, the two-state objective Γ_2 to second order in ε is

$$\Gamma_2 = \Gamma_1 + \max_{\phi,\varepsilon_1,\varepsilon_2}\left\{\frac{\Gamma_1''}{2}(\varepsilon_1^2\phi + \varepsilon_2^2(1-\phi)) \right. \\ \left. + \frac{\phi(1-\phi)F}{2}(\varepsilon_2-\varepsilon_1)^2\right\}, \tag{D9}$$

where $\Gamma_1'' = \gamma'' + \Pi(\ell_0)$ is the second derivative of the single-state objective at $\ell = \ell_0$, $F = F(\ell_0)$ and the optimization has constraints $0 < \phi < 1$ and $|\varepsilon_1|, |\varepsilon_2| \le \varepsilon$. Moreover, $\varepsilon_1 \ne \varepsilon_2$ (since otherwise that would be a single-state solution).

Taking the maximum over ϕ in Eq. D9, we get for the optimum ϕ^*,

$$\phi^* = \frac{1}{2}\left(1 - \frac{\Gamma_1''}{F}\cdot\frac{\varepsilon_2+\varepsilon_1}{\varepsilon_2-\varepsilon_1}\right). \tag{D10}$$

Note that $\Gamma_1'' < 0$ since ℓ_0 is an interior point that attains the maximum of the single-state objective. Denote $A = -\Gamma_1''/F > 0$ (since $F > 0$). Plugging ϕ^* into Eq. D9 and simplifying, we get

$$\Gamma_2 = \Gamma_1 + \frac{F}{8}\max_{\varepsilon_1,\varepsilon_2}\left\{(\varepsilon_2-\varepsilon_1)^2 - 2A(\varepsilon_2^2+\varepsilon_1^2) + A^2(\varepsilon_2+\varepsilon_1)^2\right\}, \tag{D11}$$

with the constraints $\varepsilon_1 \ne \varepsilon_2$ and $|\varepsilon_1|, |\varepsilon_2| \le \varepsilon$. There are three possibilities for the optimal $\varepsilon_1, \varepsilon_2$: (1) they are both interior points, i.e., they belong to the open interval $(-\varepsilon, \varepsilon)$, (2) one of them takes a boundary value of $\pm\varepsilon$ and the other is an interior point, or (3) both of them are at the boundary, in which case without loss of generality $\varepsilon_2 = -\varepsilon_1 = \varepsilon$ (since they cannot be equal). We now consider each case separately.

If they are both interior points, we can take the derivatives of Eq. D11 w.r.t ε_1 and ε_2 and equate them to zero. We get

$$2(\varepsilon_2^*-\varepsilon_1^*) + 2A^2(\varepsilon_1^*+\varepsilon_2^*) - 4A\varepsilon_2^* = 0, \tag{D12}$$
$$-2(\varepsilon_2^*-\varepsilon_1^*) + 2A^2(\varepsilon_1^*+\varepsilon_2^*) - 4A\varepsilon_1^* = 0. \tag{D13}$$

Solving for the two we get $\varepsilon_1^* = \varepsilon_2^*$, which violates the $\varepsilon_1 \ne \varepsilon_2$ constraint.

bioRxiv preprint doi: https://doi.org/10.1101/2025.11.02.686100; this version posted November 11, 2025. The copyright holder for this preprint (which was not certified by peer review) is the author/funder, who has granted bioRxiv a license to display the preprint in perpetuity. It is made available under aCC-BY 4.0 International license.

The second case is that one of them is on the boundary, say ε_2^*. Then, taking the derivative of Eq. D11 w.r.t ε_1 and setting it to zero, we get

$$\varepsilon_1^* = \frac{1-A}{1+A}\varepsilon_2^*. \tag{D14}$$

The term being maximized in (34) is then

$$-\frac{4A(1-A)^2}{(1+A)^2}(\varepsilon_2^*)^2 < 0, \tag{D15}$$

since $A > 0$. The single-state solution outcompetes the best two-state solution in this scenario.

This leaves the third case that $\varepsilon_2 = -\varepsilon_1 = \varepsilon$, which gives for the term being maximized in Eq. D11 as $4(1-A)\varepsilon^2$. Note that $\phi^* = 1/2$ in this case which satisfies the constraint $0 \leq \phi \leq 1$. When $A < 1$, the two-state solution outcompetes the single-state solution. Thus,

$$\Gamma_2 = \Gamma_1 + \frac{F}{2}\max\{0, (1-A)\varepsilon^2\}. \tag{D16}$$

The transition from a single-state to two-state solution occurs at $A = 1$, or in terms of the trade-off and switching statistics,

$$\left.\frac{d^2\gamma_P}{d\ell^2}\right|_{\ell_0} + \Pi(\ell_0) + F(\ell_0) = 0. \tag{D17}$$

Note that this is a sufficient condition, but not a necessary one.

a. Test for critical curvature. For a chosen growth-lag trade-off with form $\gamma_P(\ell) = \gamma_{\max} - (\gamma_{\max} - \gamma_{\min})\left(\frac{\ell_{\min}}{\ell}\right)^\alpha$, we identify the single-phenotype optimum (ℓ_0, γ_0). At this point, we construct small parabolas with varying curvatures to test when the solution transitions from a single-state solution to a two-state solution. After performing a rotation, the parabola with vertex (ℓ_0, γ_0) is given by

$$(-\sin\theta)(\ell-\ell_0) + \cos\theta(\gamma-\gamma_0) = \chi\Big[\cos\theta(\ell-\ell_0) + \sin\theta(\gamma-\gamma_0)\Big]^2. \tag{D18}$$

Taylor expanding γ around γ_0 and substituting in the equation above, we get

$$(-\sin\theta)(\ell-\ell_0) + \cos\theta\left(\gamma'(\ell-\ell_0) + \frac{\gamma''}{2}(\ell-\ell_0)^2\right) = \chi(\ell-\ell_0)^2\left[\cos\theta + \gamma'\sin\theta\right]^2. \tag{D19}$$

. Since $\gamma' = \sin\theta/\cos\theta$, this equation reduces to

$$\gamma'' = \frac{2\chi}{\cos^3\theta}. \tag{D20}$$

In the previous section, we derived the condition for the transition from a single-state solution to a two-state solution, $-[\gamma'' + \Pi(\ell_0)]/F = 1$. Assuming $\Pi(t) = e^{-t}$, the condition becomes

$$\gamma'' = 2e^{-\ell_0}. \tag{D21}$$

Finally, by equating Eq. D20 and Eq. D21, and noting that $e^{-\ell_0} = \tan\theta$, we get a relation for critical curvature parameterized by χ_{crit} for the transition

$$\chi_{\text{crit}} = \sin\theta\cos^2\theta. \tag{D22}$$

We confirmed this result numerically which is described in Fig. S3a,b.

b. Power-law growth-lag trade-off and exponential dwell times in NP. When $\gamma(\ell) = -\ell^{-\alpha}$ and $\Pi(\ell) = \frac{e^{-\ell/\eta}}{\eta}$ (recall $\eta = \gamma'/\omega'$), ℓ_0 is obtained by solving

$$\frac{\alpha}{\ell_0^{1+\alpha}} = e^{-\ell_0/\eta}. \tag{D23}$$

Using Eq. D17, we have

$$-\frac{\alpha(\alpha+1)}{\ell_0^{\alpha+2}} + \left(1 + \frac{1}{\eta}\right)e^{-\ell_0/\eta} > 0, \tag{D24}$$

which gives

$$\alpha < \left(1 + \frac{1}{\eta}\right)\ell_0 - 1. \tag{D25}$$

Plugging back $\eta = \gamma'/\omega'$ and reintroducing units, $\ell_0 \to \gamma'\ell_0$, we have

$$\alpha < (\gamma' + \omega')\ell_0 - 1. \tag{D26}$$

2. Growth–death paradigm

We now consider a similar analysis for the growth-death paradigm. We analyze two cases that are distinguished by the sign of the curvature of the growth-death trade-off. In the first case, we show that the optimal phenotype distribution *always* consists of at most two specialist phenotypes, regardless of the distribution of dwell-times in NP. In the second case, analogous to our analysis in the growth-lag scenario, we obtain a sufficient condition on the curvature of the growth-death trade-off that favor a heterogeneous, specialist strategy compared to a generalist strategy.

Let R denote the set of physiologically plausible γ, ξ values (recall that the $\gamma \geq 0, \xi \leq 0$). We define the boundary ∂R of this region as those phenotypes for which there is no other phenotype in R where both γ and ξ are larger.

bioRxiv preprint doi: https://doi.org/10.1101/2025.11.02.686100; this version posted November 11, 2025. The copyright holder for this preprint (which was not certified by peer review) is the author/funder, who has granted bioRxiv a license to display the preprint in perpetuity. It is made available under aCC-BY 4.0 International license.

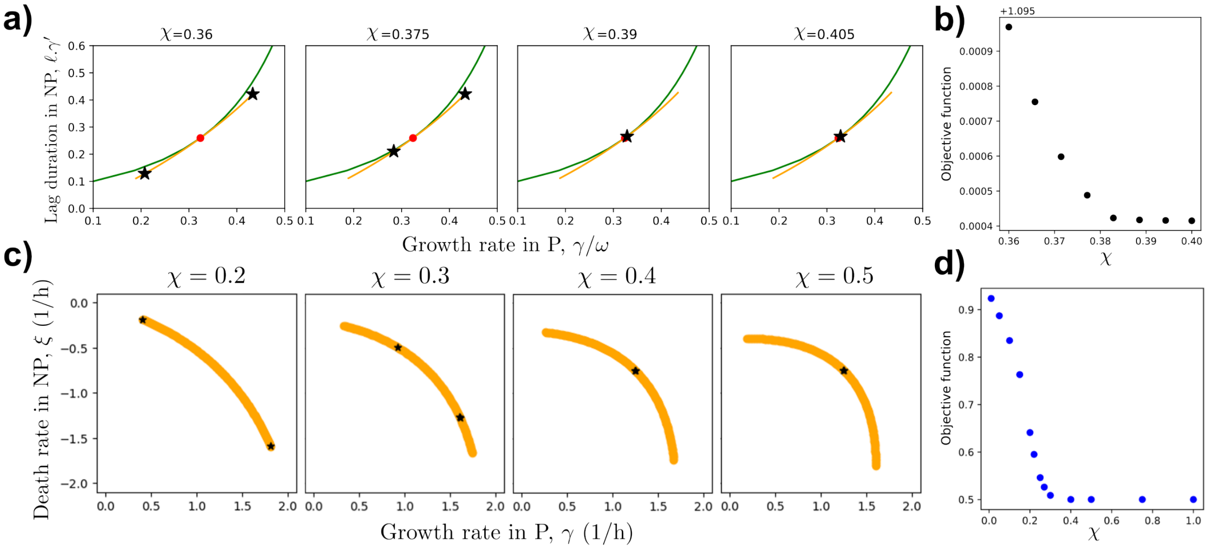

Fig. S3. Transition from a two-state solution to a single-state solution in the growth-lag system (a) and (b), and growth-death system (c) and (d). (a) The green curve represents the original power-law growth-lag trade-off for which the single-phenotype optimum, (γ_0, ℓ_0), is represented by the red circle. Taking this point as the vertex, we construct small parabolas with varying curvatures parameterized by χ and numerically determine the optimal solution. For a specific value of $\chi = \chi_{\rm crit}$, there is a transition from a two-state solution to a single-state solution. For the example shown above, this transition occurs close to the analytic prediction of $\chi_{\rm crit} = 0.3822$. (b) Maximum long-term population growth rate estimate for parabolic trade-offs with different curvatures, χ in growth-lag system. (c) Analogous to (a): Optimal solutions for different small parabola growth-death trade-offs with varying χ are tested. The analytic prediction for $\chi_{\rm crit}$ is 0.3535 for this example. (d) Maximum long-term population growth rate estimate for parabolic trade-offs with different curvatures, χ in growth-death system.

Case 1

We first consider R such that there are no physiologically plausible phenotypes on the line joining any two phenotypes on ∂R. In this case, we can show that the optimal solution is at most bimodal, regardless of the distribution over intervals in the NP environment.

Consider K phenotypes on ∂R such that $\gamma_1 > \gamma_2 > \cdots > \gamma_K$ and $\xi_1 < \xi_2 < \cdots < \xi_K$. We have

$$\Gamma(\boldsymbol{\phi}) = \sum_k \gamma_k \phi_k + \int_0^\infty dt\, \Pi(t) \log\left(\sum_k \phi_k e^{\xi_k t}\right), \quad \text{(D27)}$$

where $\Pi(t)$ is the distribution over intervals in NP. Say $\sum_k \gamma_k \phi_k = x$. We will show that for each x that is feasible, the optimal solution is a mixture of the 1st and Kth phenotypes.

The feasible points given $\sum_k \gamma_k \phi_k = x$ is the convex hull formed by points on the edges of the probability simplex. Let the vertices of the convex hull be $\boldsymbol{q}_1, \boldsymbol{q}_2, \ldots, \boldsymbol{q}_L$. Any feasible point ϕ (given x) can be written as $\sum_\mu u_\mu \boldsymbol{q}_\mu$ where $u_\mu \geq 0$, $\sum_\mu u_\mu = 1$. Consider the vector $\boldsymbol{w}(t)$ whose kth component is $w_k(t) = e^{\xi_k t}$. The last term in Eq. D27 can then be written as $\int_0^\infty dt\, \Pi(t) \log\big(\sum_\mu u_\mu \boldsymbol{q}_\mu \cdot \boldsymbol{w}(t)\big)$.

Whenever the hyperplane $\sum_k \gamma_k \phi_k = x$ intersects the probability simplex ($\gamma_1 \geq x \geq \gamma_K$), it always intersects the edge joining the vertex $\phi_1 = 1$ and the vertex $\phi_k = 1$. Say this point of intersection is $\boldsymbol{q}_1$. We have

$$\boldsymbol{q}_1 \cdot \boldsymbol{w}(t) = \frac{x - \gamma_K}{\gamma_1 - \gamma_K} e^{\xi_1 t} + \frac{\gamma_1 - x}{\gamma_1 - \gamma_K} e^{\xi_K t}. \quad \text{(D28)}$$

We will show that $\boldsymbol{q}_1 \cdot \boldsymbol{w}(t) \geq \boldsymbol{q}_\mu \cdot \boldsymbol{w}(t)$ for all μ and t. This implies that for every feasible x

$$\Gamma(\phi) \leq x + \int_0^\infty dt\, \Pi(t) \log\big(\boldsymbol{q}_1 \cdot \boldsymbol{w}(t)\big), \quad \text{(D29)}$$

with equality when $u_1 = 1$, i.e. when all probability mass is on the 1st and Kth phenotypes.

To prove $\boldsymbol{q}_1 \cdot \boldsymbol{w}(t) \geq \boldsymbol{q}_\mu \cdot \boldsymbol{w}(t)$, first consider an intermediate phenotype i with growth rates γ_i, ξ_i. Let $\boldsymbol{q}_\mu$ be the intersection of the hyperplane with the line joining $\phi_1 = 1$ and $\phi_i = 1$ ($\gamma_1 \geq x \geq \gamma_i$):

$$\boldsymbol{q}_\mu \cdot \boldsymbol{w}(t) = \frac{x - \gamma_i}{\gamma_1 - \gamma_i} e^{\xi_1 t} + \frac{\gamma_1 - x}{\gamma_1 - \gamma_i} e^{\xi_i t}. \quad \text{(D30)}$$

A short calculation gives

$$\boldsymbol{q}_1 \cdot \boldsymbol{w}(t) - \boldsymbol{q}_\mu \cdot \boldsymbol{w}(t) = \frac{\gamma_1 - x}{\gamma_1 - \gamma_i} \Bigg[\left(\tfrac{\gamma_i - \gamma_K}{\gamma_1 - \gamma_K}\right) e^{\xi_1 t} + \left(\tfrac{\gamma_1 - \gamma_i}{\gamma_1 - \gamma_K}\right) e^{\xi_K t} - e^{\xi_i t} \Bigg]. \quad \text{(D31)}$$

Since e^t is convex, one obtains

$$\boldsymbol{q}_1 \cdot \boldsymbol{w}(t) - \boldsymbol{q}_\mu \cdot \boldsymbol{w}(t) \geq \frac{\gamma_1 - x}{\gamma_1 - \gamma_i} \big(e^{\beta t} - e^{\xi_i t}\big), \quad \text{(D32)}$$

bioRxiv preprint doi: https://doi.org/10.1101/2025.11.02.686100; this version posted November 11, 2025. The copyright holder for this preprint (which was not certified by peer review) is the author/funder, who has granted bioRxiv a license to display the preprint in perpetuity. It is made available under aCC-BY 4.0 International license.

where

$$\beta = \frac{\gamma_i - \gamma_K}{\gamma_1 - \gamma_K}\,\xi_1 + \frac{\gamma_1 - \gamma_i}{\gamma_1 - \gamma_K}\,\xi_K. \tag{D33}$$

Thus $\boldsymbol{q}_1 \cdot \boldsymbol{w}(t) \geq \boldsymbol{q}_\mu \cdot \boldsymbol{w}(t)$ if

$$\frac{\xi_1 - \xi_K}{\gamma_1 - \gamma_K} \geq \frac{\xi_i - \xi_K}{\gamma_i - \gamma_K}. \tag{D34}$$

By the assumed shape of ∂R, this holds for all i. A similar argument applies when the hyperplane intersects the edge joining $\phi_k = 1$ and $\phi_i = 1$ ($\gamma_K \leq x \leq \gamma_i$). Hence $\boldsymbol{q}_1 \cdot \boldsymbol{w}(t) \geq \boldsymbol{q}_\mu \cdot \boldsymbol{w}(t)$ for every μ.

Case 2

Now suppose ∂R does not satisfy the conditions of Case 1, so solutions with more than two modes become possible. Nonetheless, we can state when more than one phenotype is favored. The best single-phenotype strategy corresponds to the point where a line of slope -1 is tangent to ∂R in the (γ, ξ) plane. If the curvature of ∂R near this point is small enough, a heterogeneous strategy outcompetes the single-phenotype strategy.

To see this, consider an infinitesimal parabola $w = \chi z^2$ around the best phenotype (γ, ξ). The z-axis is along the tangent; the w-axis points away and downward. We look at solutions on this parabola with $|z| \leq \varepsilon \ll 1$ for various χ.

As in Case 1,

$$\Gamma(\boldsymbol{\phi}) = \sum_k \gamma_k \phi_k + \int_0^\infty dt\, \Pi(t) \log\left(\sum_k \phi_k e^{\xi_k t}\right). \tag{D35}$$

Rescale t and ξ_k so that $\langle t \rangle = 1$. Since solutions are at most bimodal, let one phenotype have probability ϕ and lie at z_1, the other at z_2. Writing $\gamma_i = \gamma + \Delta\gamma_i$ and $\xi_i = \xi + \Delta\xi_i$ for $i = 1, 2$, a second-order Taylor expansion gives

$$\Gamma(\phi) - \gamma - \xi \approx \Gamma_\varepsilon(\phi) = \phi\mu_1 + (1-\phi)\mu_2 - \frac{\tau_2^2}{2}\left(\phi\nu_1 + (1-\phi)\nu_2\right)^2, \tag{D36}$$

with $\tau_2^2 = \langle t^2 \rangle$, $\mu_i = \Delta\gamma_i + \Delta\xi_i + \tau_2^2(\Delta\xi_i)^2/2$ and $\nu_i = \Delta\xi_i$. Optimizing over ϕ yields

$$\phi^* = \frac{\mu_1 - \mu_2}{\tau_2^2(\nu_1 - \nu_2)^2} - \frac{\nu_2}{\nu_1 - \nu_2}, \tag{D37}$$

for $0 \leq \phi^* \leq 1$. Substituting back,

$$\Gamma_\varepsilon(\phi^*) = \frac{(\mu_1 - \mu_2)^2}{2\tau_2^2(\nu_1 - \nu_2)^2} + \frac{\mu_2\nu_1 - \mu_1\nu_2}{\nu_1 - \nu_2}. \tag{D38}$$

With $\Delta\gamma_i = (z_i - \chi z_i^2)/\sqrt{2}$ and $\Delta\xi_i = -(z_i + \chi z_i^2)/\sqrt{2}$ and retaining terms up to second order,

$$\Gamma_\varepsilon(\phi^*) \approx \frac{\kappa^2(z_1 + z_2)^2}{\tau_2^2} - \kappa z_1 z_2, \tag{D39}$$

where $\kappa = \tau_2^2/4 - \sqrt{2}\,\chi$.

For $\kappa < 0$, $a > 1$ and the optimum reduces to the single best phenotype; in this case,

$$\Gamma_\varepsilon(\phi^*) \approx \frac{\kappa^2 z_1^2}{\tau_2^2}\left(\frac{1 - a^2}{a^2}\right) < 0, \tag{D40}$$

which is suboptimal.

If $\kappa > 0$, a bimodal solution with $z_1 z_2 < 0$ beats the single-phenotype solution (provided $0 \leq \phi^* \leq 1$). One shows the optimum occurs at $z_1 = -z_2 = \varepsilon$:

$$\Gamma_\varepsilon(\phi^*) \approx \frac{\kappa^2 z_1^2}{\tau_2^2}\left((a + x)^2 + 1 - a^2\right), \tag{D41}$$

where $x = z_2/z_1$ and $a = 1 - \tau_2^2/2\kappa$. Since $a < -1$ when $\kappa > 0$, $x = -1$ maximizes Eq. D41. Then $\phi^* = 1/2$ and $\Gamma_\varepsilon(p^*) \approx \kappa z_1^2 > 0$, maximized at $z_1 = \varepsilon$.

Hence, a heterogeneous solution is favored if $\kappa > 0$, i.e.,

$$\chi < \frac{\langle t^2 \rangle}{4\sqrt{2}}, \tag{D42}$$

where $\langle t^2 \rangle$ is the second moment of the NP dwell-time distribution. If the curvature χ of ∂R is below this threshold, a mixed two-phenotype strategy (specialists) outperforms the single-phenotype strategy (generalist). This is confirmed numerically where we plot the maximum long-term growth rate for different χ values as shown in Fig. S3d. For χ above the threshold, the optimal growth rate stays constant as the single phenotype solution doesn't change with χ. For χ below the threshold, we see that higher values for the long-term growth are achievable with two-phenotype strategies.

We chose parabolic curves centered at a specific point with different curvatures χ for the growth-death trade-off. In Fig. S3c, as χ increases, the optimal solution transitions from a bimodal phenotype distribution to a single-phenotype distribution.

Appendix E: Two-phenotype system in the growth-lag paradigm

1. Why do arresters have infinite lag?

Assuming that the population can have two phenotypes with lags ℓ_1 and ℓ_2 such that $\ell_{min} \leq \ell_1 < \ell_2$ and the fraction of the population with lag ℓ_1 in P is ϕ. We consider growth-lag trade-offs such that the growth rate in P of a cell with lag ℓ in NP is

$$\gamma_{\mathrm{P}}(\ell) = \gamma_{\max} - (\gamma_{\max} - \gamma_{\min})\left(\frac{\ell_{\min}}{\ell}\right)^\alpha. \tag{E1}$$

Consider the term to be maximized over ϕ in Eq. B15, which we denote as $\Gamma(\phi, \ell_1, \ell_2)$. We consider $\Pi(t) =$

bioRxiv preprint doi: https://doi.org/10.1101/2025.11.02.686100; this version posted November 11, 2025. The copyright holder for this preprint (which was not certified by peer review) is the author/funder, who has granted bioRxiv a license to display the preprint in perpetuity. It is made available under aCC-BY 4.0 International license.

$\omega' e^{-\omega' t}$. Interchanging the integrals over τ and t, we get

$$\Gamma(\phi,\ell_1,\ell_2) = \phi\gamma_{\mathrm{P}}(\ell_1)+(1-\phi)\gamma_{\mathrm{P}}(\ell_2)+\omega\int_0^{\infty} e^{-\omega' t}\overline{\gamma}_{\mathrm{NP}}(t)dt, \tag{E2}$$

where $\overline{\gamma}_{\mathrm{NP}}(t)$ is the average growth rate at time t after the environment is switched from P to NP given that the population began with fraction ϕ cells in phenotype 1. Specifically,

$$\overline{\gamma}_{\mathrm{NP}}(t) = \gamma' \begin{cases} 0 & t < \ell_1 \\ \frac{\phi e^{\gamma'(t-\ell_1)}}{\phi e^{\gamma'(t-\ell_1)}+(1-\phi)} & \ell_1 < t \le \ell_2 \\ 1 & t \ge \ell_2 \end{cases} \tag{E3}$$

Rescaling time: $t \to \gamma' t$, and defining $\eta = \gamma'/\omega'$, we have

$$\Gamma(\phi,\ell_1,\ell_2) = \max_{\phi}\Bigg\{\phi\gamma_{\mathrm{P}}(\ell_1) + (1-\phi)\gamma_{\mathrm{P}}(\ell_2) + \omega\left(\int_{\ell_1}^{\ell_2} dt e^{-\frac{t}{\eta}}\frac{\phi e^{t-\ell_1}}{\phi e^{t-\ell_1}+(1-\phi)} + e^{-\frac{\ell_2}{\eta}}\right)\Bigg\}. \tag{E4}$$

Shifting time as $t \to t-\ell_1$ and after a few simplifications, Γ reduces to the objective function

$$f(\phi,\ell_1,\ell_2) = -\frac{\phi}{\ell_1^{\alpha}} - \frac{1-\phi}{\ell_2^{\alpha}} + ke^{-\frac{\ell_1}{\eta}}\left[\int_0^x dt e^{-\frac{t}{\eta}}\frac{\phi e^t}{\phi e^t + (1-\phi)} + \int_{\ell_2-\ell_1}^{\infty} dt e^{-\frac{t}{\eta}}\frac{1-\phi}{\phi e^t+(1-\phi)}\right] \tag{E5}$$

where k is a positive constant obtained by absorbing ω with $(\gamma_{\max}-\gamma_{\min})\ell_{\min}^{\alpha}$, and $\ell_2 = \ell_1 + x$. Substituting $\phi = \frac{1}{1+e^{-\beta}}$, we derive an equivalent form for the objective function

$$f(\beta,\ell_1,x) = -\frac{1}{(1+e^{-\beta})\ell_1^{\alpha}} - \frac{e^{-\beta}}{(1+e^{-\beta})(\ell_1+x)^{\alpha}} + ke^{-\frac{\ell_1}{\eta}}\left[\int_0^x dt e^{-\frac{t}{\eta}}\frac{e^t}{e^t+e^{-\beta}} + \eta e^{-\frac{x}{\eta}}\right]. \tag{E6}$$

Taking the derivative of f with respect to x yields

$$\frac{\partial f}{\partial x} = \frac{\alpha e^{-\beta}}{(1+e^{-\beta})(\ell_1+x)^{\alpha+1}} - ke^{-\frac{\ell_1+x}{\eta}}\left(\frac{e^{-\beta}}{e^x+e^{-\beta}}\right) \tag{E7}$$

The first term on the right hand side is always positive and decays with x as a power-law, whereas the second term is always negative and decays exponentially. For sufficiently large x, the first term dominates and the gradient is positive. That is, it is preferable to indefinitely increase ℓ_2. While this argument does not prove that the arrester solution is the preferred phenotype under all scenarios, it helps rationalize why an arrester phenotype with an infinite lag is selected.

2. Relating the lag duration of recoverers and the fraction of recoverers

We now consider a situation where the population can choose the fraction of recoverers ϕ and their lag duration $\ell \ge \ell_{\min}$ (and consequently their growth rate $\gamma(\ell)$). This scenario corresponds to $\ell_1 = \ell$ and $\ell_2 = \infty$ from Section E 1. The average growth in NP $\overline{\gamma}_{\mathrm{NP}}(t)$ satisfies $\overline{\gamma}_{\mathrm{NP}}(t) = 0$ for $t \le \ell$. For $t > \ell$, we have

$$\overline{\gamma}_{\mathrm{NP}}(t) = \gamma'\frac{\phi e^{\gamma'(t-\ell)}}{1-\phi+\phi e^{\gamma'(t-\ell)}}. \tag{E8}$$

Substituting $t \to t-\ell$ and simplifying, we obtain

$$V(\phi,\ell) = \gamma' e^{-\omega'\ell}\int_0^{\infty} dt\,\frac{(1-\phi)e^{-\omega' t}}{(1-\phi)+\phi e^{-\gamma' t}}. \tag{E9}$$

The integral on the right-hand side can be expressed in terms of the hypergeometric function ${}_2F_1(a,b;c;x)$. Denoting $\eta \equiv \gamma'/\omega'$, we have

$$V(\phi,\ell) = \eta\, e^{-\omega'\ell}\,{}_2F_1\left(1,\frac{1}{\eta};\frac{1}{\eta}+1;-\frac{1-\phi}{\phi}\right). \tag{E10}$$

When $0 < \phi < 1$ and $\ell > \ell_{\min}$, we can optimize for ϕ and ℓ by taking the derivative of the growth function and setting it to zero. For a population in state s in P, we obtain the optimality relation

$$\frac{\partial V}{\partial\phi}\Big/\frac{\partial\overline{\gamma}_{\mathrm{P}}}{\partial\phi} = \frac{\partial V}{\partial\ell}\Big/\frac{\partial\overline{\gamma}_{\mathrm{P}}}{\partial\ell} = -\frac{1}{\omega(s)}. \tag{E11}$$

The first equation above expresses a relationship between the optimal ℓ^* and ϕ^* independent of $\omega(s)$. We then obtain,

$$\ell^* = \frac{\alpha\,\eta\,{}_2F_1\left(2,\frac{1}{\eta}+1;\frac{1}{\eta}+2;-\frac{1-\phi^*}{\phi^*}\right)}{\gamma'\phi^*(\eta+1)\,{}_2F_1\left(1,\frac{1}{\eta};\frac{1}{\eta}+1;-\frac{1-\phi^*}{\phi^*}\right)} \tag{E12}$$

Appendix F: Switching between two phenotypes in the preferred environment

While setting up the model, we made an assumption that cells rapidly switch between different phenotypes to maintain a constant phenotype distribution in the preferred environment (P). This switching occurs on timescales shorter that any doubling time and typical time spent in P, but slower than the time it takes to switch between P and NP. This ensures that cells cannot adapt while the environment is switching. In this section we illustrate the balance between growth and switching that ensures a constant phenotype distribution in P.

Suppose the population is composed of two phenotypes with growth rates γ_1 and γ_2 ($\gamma_2 > \gamma_1$). N_{tot} is the total number of initial cells of which N_1 cells adopt phenotype

bioRxiv preprint doi: https://doi.org/10.1101/2025.11.02.686100; this version posted November 11, 2025. The copyright holder for this preprint (which was not certified by peer review) is the author/funder, who has granted bioRxiv a license to display the preprint in perpetuity. It is made available under aCC-BY 4.0 International license.

1 and $N_2(= N_{\text{tot}} - N_1)$ cells adopt phenotype 2. The time-evolution of N_{tot} and N_1 are governed by

$$\frac{dN_{\text{tot}}}{dt} = \gamma_1 N_1 + \gamma_2(N_{\text{tot}} - N_1), \tag{F1}$$

$$\frac{dN_1}{dt} = \gamma_1 N_1 + \psi_{21}(N_{\text{tot}} - N_1) - \psi_{12} N_1 \tag{F2}$$

where ψ_{21} and ψ_{12} are the switching rates from phenotypes 2 to 1 and 1 to 2 respectively. The quantity that remains constant in the preferred environment is $\phi = N_1/N_{\text{tot}}$ such that $d\phi/dt = 0$. This gives us,

$$\frac{d\phi}{dt} = \frac{d(N_1/N_{\text{tot}})}{dt} = \frac{N_{\text{tot}}(dN_1/dt) - N_1(dN_{\text{tot}}/dt)}{N_{\text{tot}}^2} = 0. \tag{F3}$$

Substituting Eq. F1 and Eq. F2 and after simplifying we get,

$$\phi^2 - (1 + \hat{\psi}_{21} + \hat{\psi}_{12})\phi + \hat{\psi}_{21} = 0, \tag{F4}$$

where we have defined $\hat{\psi}_{21} = \psi_{21}/(\gamma_2 - \gamma_1), \hat{\psi}_{12} = \psi_{12}/(\gamma_2 - \gamma_1)$. The solution for ϕ is given by

$$\phi = \frac{(1 + \hat{\psi}_{21} + \hat{\psi}_{12}) \pm \sqrt{(1 + \hat{\psi}_{21} + \hat{\psi}_{12})^2 - 4\hat{\psi}_{21}}}{2}. \tag{F5}$$

Tuning $\hat{\psi}_{21}$ and $\hat{\psi}_{12}$ allows for tuning ϕ to any value between 0 and 1. For example, when the rate of switching is much larger than the difference in growth rates, i.e., $\hat{\psi}_{21}, \hat{\psi}_{12} \gg 1$, we can approximate the steady-state ϕ using Eq. F5 as

$$\phi \approx \frac{\hat{\psi}_{21}}{\hat{\psi}_{21} + \hat{\psi}_{12}}. \tag{F6}$$

The sum $\hat{\psi}_{21} + \hat{\psi}_{12}$ sets the rate at which the population reaches steady-state, whereas the ratio $\hat{\psi}_{21}/\hat{\psi}_{12}$ sets the value of ϕ at this steady-state.

Appendix G: A minimal metabolic model illustrating constraints on speed of adaptation

Microorganisms encounter environments in which the available carbon source fluctuates between preferred and non-preferred nutrients. Certain nutrients (e.g. glucose) support rapid growth by activating the corresponding metabolic pathways (e.g. glycolysis), whereas other nutrients (e.g. glycerol, acetate) require induction of alternative pathways (e.g. gluconeogenesis). This results in a characteristic lag phase prior to growth resumption. Fast growth on the preferred nutrient and rapid adaptation (short lag) on the non-preferred nutrient are inherently antagonistic, reflecting a growth–lag trade-off. We present a minimal model, inspired by central metabolism, which captures a growth-lag trade-off that could arise due to antagonism in core pathways.

Model structure and assumptions

The schematic of the model is shown in Fig. 4a, where:

- G and N denote the concentrations of the metabolite pools in the preferred environment (P) and non-preferred environment (NP), respectively.
- g and n denote intracellular concentrations of the central metabolites.
- r is the maximal forward flux rate of the irreversible reactions in both P and NP.
- δ is the loss rate constant for g and n.
- φ and ζ are the abundances of the irreversible enzymes that catalyze the forward ($g \to n$) and reverse ($n \to g$) reactions, respectively.

We assume Michaelis–Menten kinetics for each enzymatic step. Growth rate linked to biomass production, μ, is assumed to depend on the levels of the metabolites g and n:

$$\mu = \left(\frac{n}{n + K_{\text{BM}}}\right)^{\nu} \times \left(\frac{g}{g + K_{\text{BM}}}\right)^{\nu} \tag{G1}$$

where K_{BM} is the half-saturation constant, and the exponent $\nu > 0$ captures effective cooperative mechanisms in metabolism.

The time evolution of each metabolite pool is given by mass-action and Michaelis–Menten type terms. In the preferred environment when G is present and N is absent, the equations are

$$\begin{aligned}\frac{dg}{dt} &= rG - \varphi\frac{g}{K_g + g} + \zeta\frac{n}{K_n + n} - \nu\mu \\ &\quad - \delta g, \\ \frac{dn}{dt} &= \varphi\frac{g}{K_g + g} - \zeta\frac{n}{K_n + n} - \nu\mu - \delta n.\end{aligned}$$

In the non-preferred environment when G is absent and N is present, the equations are

$$\begin{aligned}\frac{dg}{dt} &= -\varphi\frac{g}{K_g + g} + \zeta\frac{n}{K_n + n} - \nu\mu - \delta g, \\ \frac{dn}{dt} &= rN + \varphi\frac{g}{K_g + g} - \zeta\frac{n}{K_n + n} - \nu\mu \\ &\quad - \delta n.\end{aligned}$$

We fix the concentrations of G and N to be 2 a.u. and 1 a.u. in the preferred and non-preferred environments, respectively.

bioRxiv preprint doi: https://doi.org/10.1101/2025.11.02.686100; this version posted November 11, 2025. The copyright holder for this preprint (which was not certified by peer review) is the author/funder, who has granted bioRxiv a license to display the preprint in perpetuity. It is made available under aCC-BY 4.0 International license.

Enzyme dynamics and regulation

We model the synthesis and dilution of the irreversible enzymes, φ and ζ, by simple first-order kinetics toward nutrient-specific steady-state levels:

$$\frac{d\varphi}{dt} = \mu\big(\varphi_{\rm ss}^{(\rm nutrient)} - \varphi\big)\,, \qquad \text{(G2)}$$
$$\frac{d\zeta}{dt} = \mu\big(\zeta_{\rm ss}^{(\rm nutrient)} - \zeta\big)\,, \qquad \text{(G3)}$$

where

- μ is the instantaneous growth rate;
- $\varphi_{\rm ss}^{(\rm nutrient)}$ and $\zeta_{\rm ss}^{(\rm nutrient)}$ are the steady-state enzyme levels where the 'nutrient' may be G or N;
- $\varphi_{\rm ss}^G > \zeta_{\rm ss}^G$ biases flux in the direction $g \to n$ when G is the nutrient, and vice versa after a nutrient shift to N being the nutrient.

Origin of the power-law growth–lag trade-off

Upon a sudden shift from P to NP, the pre-existing high level of φ suppresses flux from $n \to g$ and dissipates excess accumulation of n. This depletes cellular resources and delays the reversal of net flux. Because φ decays only on the timescale $\sim 1/\mu$, flux reversal stalls until φ falls and ζ rises toward $\zeta_{\rm ss}^N$ so that the growth rate μ can rise. This leads to a lag before growth can resume.

The metabolic model operates in at least three qualitatively distinct regimes, delineated by $\varphi_{\rm ss}^G$, depending on how the system produces new enzymes ζ after the switch from P to NP. Here, we expand on the third scenario discussed in the main text, where $\varphi_{\rm ss}^G$ has intermediate values such that the value of $\zeta_{\rm ss}^G$ that maximizes pre-shift growth rate is zero (Fig. S4). We show below that the growth-lag trade-off in this regime is a power-law with exponent $1/(\nu - 1)$.

Suppose $\varphi = \varphi_{\rm ss}^G$ and $\zeta = \zeta_{\rm ss}^G$ are the steady state values of the enzymes in P. At steady-state, g and n are found by solving

$$G - \varphi v_1(g) + \zeta v_2(n) - \delta g - \nu\mu(g,n) = 0,$$
$$\varphi v_1(g) - \zeta v_2(n) - \delta n - \nu\mu(g,n) = 0,$$

where $r = 1$, $v_1(x) = x/(x+K)$ (assume $K_g = K_n = K$), $\mu(g,n) = (v_2(g)v_2(n))^\nu$ and $v_2(x) = x/(x+K_{\rm BM})$.

We consider a parameter regime where, given $\varphi_{\rm ss}^G$, $\mu_{\rm P}$ is maximized when $\zeta_{\rm ss}^G = 0$ and consequently ℓ is infinite. We calculate how $\mu_{\rm P}$ and ℓ decrease with $\zeta_{\rm ss}^G$ when $0 < \zeta_{\rm ss}^G \ll 1$.

The maximal growth rate when $\zeta_{\rm ss}^G = 0$ is found by solving the steady state equations

$$0 = G - \varphi_{\rm ss}^G v_1(g^*) - \delta g^* - \nu\mu(g^*, n^*),$$
$$0 = \varphi_{\rm ss}^G v_1(g^*) - \delta n^* - \nu\mu(g^*, n^*),$$

where g^*, n^* are the values at steady state in this scenario. Let $\Delta g = g - g^*$ and $\Delta n = n - n^*$ be the deviations from these values when $0 < \zeta_{\rm ss}^G \ll 1$. Δg and Δn are of order $\zeta_{\rm ss}^G$. Expanding the steady state equations to first order when $0 < \zeta_{\rm ss}^G \ll 1$, Δg and Δn satisfy

$$\zeta_{\rm ss}^G v_1(n^*) = \left(\delta + \nu\frac{\partial\mu_{\rm P}}{\partial g} + \varphi_{\rm ss}^G v_1'(g^*)\right)\Delta g + \left(\nu\frac{\partial\mu_{\rm P}}{\partial n}\right)\Delta n,$$
$$\zeta_{\rm ss}^G v_1(n^*) = \left(\varphi_{\rm ss}^G v_1'(g^*) - \nu\frac{\partial\mu_{\rm P}}{\partial g}\right)\Delta g + \left(-\delta - \nu\frac{\partial\mu_{\rm P}}{\partial n}\right)\Delta n,$$

where the derivatives are evaluated at g^*, n^*. Since $\Delta\mu_{\rm P} = \frac{\partial\mu_{\rm P}}{\partial g}\Delta g + \frac{\partial\mu_{\rm P}}{\partial n}\Delta n$ (to first order in $\zeta_{\rm ss}^G$), we solve Δg and Δn by solving the above pair of equations. This gives an expression for $\Delta\mu_{\rm P}$:

$$\Delta\mu_{\rm P} = \zeta_{\rm ss}^G \begin{pmatrix}\frac{\partial\mu_{\rm P}}{\partial g} & \frac{\partial\mu_{\rm P}}{\partial n}\end{pmatrix} A^{-1}\begin{pmatrix}v_1(n^*)\\ v_1(n^*)\end{pmatrix}, \quad \text{where}$$
$$A = \begin{pmatrix}\delta + \nu\frac{\partial\mu_{\rm P}}{\partial g} + \varphi_{\rm ss}^G v_1'(g^*) & \nu\frac{\partial\mu_{\rm P}}{\partial n}\\ \varphi_{\rm ss}^G v_1'(g^*) - \nu\frac{\partial\mu_{\rm P}}{\partial g} & -\delta - \nu\frac{\partial\mu_{\rm P}}{\partial n}\end{pmatrix} \qquad \text{(G4)}$$

which we can write as $\Delta\mu_{\rm P} = \zeta_{\rm ss}^G b$, where b is a scalar obtained from the vector-matrix-vector product on the right hand side of the above equation.

After the switch to NP (starting from the steady state in P with $0 < \zeta_{\rm ss}^G \ll 1$), we have

$$\frac{dg}{dt} = -\varphi v_1(g) + \zeta v_2(n) - \delta g - \nu\mu, \qquad \text{(G5)}$$
$$\frac{dn}{dt} = N - \varphi v_1(g) + \zeta v_2(n) - \delta n - \nu\mu, \qquad \text{(G6)}$$

where

$$\frac{d\varphi}{dt} = \mu\left(\varphi_{\rm ss}^N - \varphi\right), \qquad \text{(G7)}$$
$$\frac{d\zeta}{dt} = \mu\left(\zeta_{\rm ss}^N - \zeta\right). \qquad \text{(G8)}$$

with initial conditions $\varphi = \varphi_{\rm ss}^G$ and $\zeta = \zeta_{\rm ss}^G$. The approximations are valid soon after the switch. Dividing these two equations, we get $d\varphi/d\zeta = (\varphi_{\rm ss}^N - \varphi)/(\zeta_{\rm ss}^N - \zeta)$. Integrating, we get

$$\frac{\varphi_{\rm ss}^N - \varphi}{\varphi_{\rm ss}^N - \varphi_{\rm ss}^G} = \frac{\zeta_{\rm ss}^N - \zeta}{\zeta_{\rm ss}^N - \zeta_{\rm ss}^G}. \qquad \text{(G9)}$$

As discussed earlier, we work in the limit where after the switch, g depletes rapidly due to both the transformation from g to n catalyzed by φ, and due to loss at rate δ. In quantitative terms, the first and third terms on the rhs of (G5) contribute to the outflux of g.

During the lag phase, both φ and ζ do not change significantly from $\varphi_{\rm ss}^G$ and $\zeta_{\rm ss}^G$ as biomass production (i.e., μ) is minimal in this phase. When $\varphi_{\rm ss}^G$ is large, g drops rapidly to near zero until it reaches a steady state. Since $v_1(g) \approx g$ when $g \ll K$ and $v_1(g) \approx 1$ when $g \gg K$, either g initially decreases exponentially or linearly with rate $\varphi_{\rm ss}^G$ depending on the value of g^*. Moreover, when

bioRxiv preprint doi: https://doi.org/10.1101/2025.11.02.686100; this version posted November 11, 2025. The copyright holder for this preprint (which was not certified by peer review) is the author/funder, who has granted bioRxiv a license to display the preprint in perpetuity. It is made available under aCC-BY 4.0 International license.

$g \ll 1$, $\mu \ll v_1(g)$ as $\mu \sim g^\nu$ and $v_1(g) \sim g$ for small g and $\nu > 1$. That is, $dg/dt \approx -(\varphi + \delta)g + \zeta v_1(n)$. At steady state, we have $(\varphi + \delta)g \approx \zeta v_1(n)$.

Moreover, in steady state, from (G6) we see that all terms except N and δn are negligible so that $n \approx N/\delta$. Thus,

$$g \approx \left(\frac{\zeta}{\delta + \varphi_{\rm ss}^G}\right) v_1(N/\delta), \tag{G10}$$

where have used (G9) to eliminate φ and ignored all terms second order in ζ. Plugging this expression for g into μ in (G8), we have

$$\frac{d\zeta}{dt} \approx \zeta^\nu \left(\frac{v_1(N/\delta)v_2(N/\delta)}{\delta + \varphi_{\rm ss}^G}\right)^\nu . \tag{G11}$$

Integrating this from $\zeta = \zeta_{\rm ss}^G$ to ∞ (or some large value) for $\nu > 1$, we get an estimate of lag ℓ as the time it takes for ζ to diverge:

$$\ell \approx \frac{(\zeta_{\rm ss}^G)^{1-\nu}}{\nu - 1} \left(\frac{\delta + \varphi_{\rm ss}^G}{v_1(N/\delta)v_2(N/\delta)}\right)^\nu . \tag{G12}$$

Plugging in $\zeta_{\rm ss}^G = \Delta\mu_{\rm P}/b$, we have

$$\Delta\mu_{\rm P} = A\ell^{-\frac{1}{\nu-1}}, \tag{G13}$$

for some constant A.

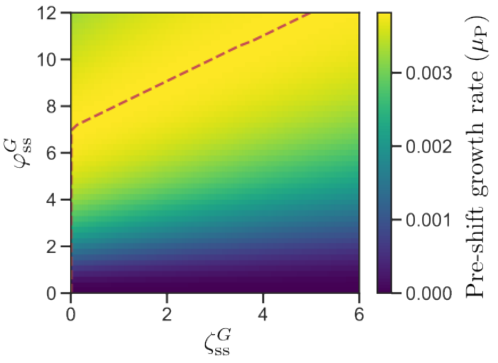

Fig. S4. Heatmap of pre-shift growth rate $\mu_{\rm P}$ for different $\varphi_{\rm ss}^G$ and $\zeta_{\rm ss}^G$ values. Other parameters in the model are fixed. Above a certain value of $\varphi_{\rm ss}^G$, the optimal pre-shift growth rate is achieved at a non-zero $\zeta_{\rm ss}^G$ (dashed red curve).
